# MtiBase: a database for decoding microRNA target sites located within CDS and 5′UTR regions from CLIP-Seq and expression profile datasets

**DOI:** 10.1093/database/bav102

**Published:** 2015-10-21

**Authors:** Zhi-Wei Guo, Chen Xie, Jian-Rong Yang, Jun-Hao Li, Jian-Hua Yang, Limin Zheng

**Affiliations:** ^1^Key Laboratory of Gene Engineering of the Ministry of Education, State Key Laboratory of Biocontrol,; ^2^Key Laboratory of Liver Disease of Guangdong Province, The Third Affiliated Hospital, Sun Yat-Sen University, Guangzhou, People’s Republic of China,; ^3^Department of Ecology and Evolutionary Biology, University of Michigan, Ann Arbor, MI, USA and; ^4^Collaborative Innovation Center for Cancer Medicine, Sun Yat-sen University Cancer Center, State Key Laboratory of Oncology in South China, Guangzhou, P. R. China

## Abstract

MicroRNAs (miRNAs) play an important role in the regulation of gene expression. Previous studies on miRNA functions mainly focused on their target sites in the 3′ untranslated regions (UTRs) of mRNAs. However, increasing evidence has revealed that miRNAs can also induce mRNA degradation and mediate translational repression via complementary interactions with the coding sequence (CDS) and 5′UTR of mRNAs. In this study, we developed a novel database, MtiBase, to facilitate the comprehensive exploration of CDS- and 5′UTR-located miRNA target sites identified from cross-linking immunoprecipitation sequencing (CLIP-Seq) datasets and to uncover their regulatory effects on mRNA stability and translation from expression profile datasets. By integrating 61 Argonaute protein-binding CLIP-Seq datasets and miRNA target sites predicted by five commonly used programs, we identified approximately 4 400 000 CDS-located and 470 000 5′UTR-located miRNA target sites. Moreover, we evaluated the regulatory effects of miRNAs on mRNA stability and translation using the data from 222 gene expression profiles, and 28 ribosome-protected fragment sequencing, and six pulsed stable isotope labeling with amino acids in culture. Finally, the effects of SNPs on the functions of miRNA target sites were systematically evaluated. Our study provides a useful tool for functional studies of miRNAs in regulating physiology and pathology.

**Database URL:** http://mtibase.sysu.edu.cn

## Introduction

MicroRNAs (miRNAs) are ∼22-nucleotide (nt) endogenous RNAs that participate in important biological processes by regulating gene expression (1). MiRNAs mediate gene expression by guiding interaction of the miRNA-induced silencing complex, composed of Argonaute proteins (Ago) and miRNAs, with target mRNAs (2). MiRNAs have been shown to induce mRNA degradation and repress translation by hybridizing with the 3′untranslated regions (UTR) of mRNAs (2).

Although the majority of functional miRNA target sites are found in the 3′UTR of mRNAs, an increasing number of functional sites have been discovered in the coding sequence (CDS) and 5′UTR of mRNAs in multiple organisms (3–8). For example, miR-2 triggers mRNA degradation and inhibits translation through its CDS- and 5′UTR-located targets in Drosophila (9). In mice, recent studies have revealed that miR-134, miR-296 and miR-470 can regulate the differentiation of embryonic stem cells via complementary interactions with the CDS of the transcription factors Nanog homeobox, POU class 5 homeobox 1 and sex-determining region Y-box 2 (10). In humans, miR-181 can hybridize with the CDS of tumor suppressors RB-associated KRAB zinc finger and retinoblastoma 1, and induce the degradation of their mRNAs (11). In addition to these target sites, cross-linking immunoprecipitation sequencing [CLIP-Seq, e.g. High-throughput sequencing together with UV (HITS)-CLIP, photoactivatable ribonucleoside-enhanced (PAR)-CLIP, iCLIP, cross-linking ligation and sequencing of hybrids (CLASH)] datasets have detected numerous Ago-binding regions within mRNA CDS and 5′UTR (12–14), which reveal the existence of numerous miRNA target sites in these regions. Meanwhile, recent studies have shown that the target sequences of CDS- and 5′UTR-located miRNA target sites are conserved (15–17) and that the CDS-located miRNA target sites are the most potent for repressing translation (18). Therefore, identifying the functional sites within mRNA CDS and 5′UTR may be of great significance in uncovering the comprehensive regulatory effects of miRNAs.

In recent years, advanced, high-throughput experimental approaches have been developed to identify miRNA target sites and to assess their regulatory effects. Ago CLIP-Seq is an approach for isolating the Ago-binding regions within mRNAs; this method significantly reduces the false positive rates of miRNA target prediction by limiting the size of search space (12, 14). Apart from CLIP-Seq, gene expression profiles detected by microarrays or RNA sequencing (RNA-seq) allow evaluation of miRNA regulatory effects on mRNA stability in response to miRNA overexpression, knockdown or knockout. Moreover, ribosome-protected fragment sequencing (RPF) (19) and pulsed stable isotope labeling with amino acids in culture (pSILAC) (20) have been used to assess the impacts of miRNAs on translational efficiency and protein synthesis, respectively. The increasing amount of experimental data has generated a great demand for a database to systematically integrate and annotate these data to facilitate the investigation of miRNAs.

Single-nucleotide polymorphisms (SNPs) are critical causes of transcript variation and are closely connected with gene expression, phenotypes and diseases (21). They are widespread in the genomes of different organisms (22). Recently, SNPs were shown to cause abnormal gene expression by disrupting the interactions between miRNAs and their CDS-located binding sites, which reveal that the regulatory effects of CDS-located miRNA target sites are significantly affected by SNPs (23). For example, the rs799917 TC genotype of human *BRCA1* promotes the development of gastric cancer as a result of disruption of the CDS-located miRNA–target interactions (23). Moreover, CDS-located target sites can act as a surveillance system that induces the degradation of the aberrant transcripts to maintain the normal function of an organism (24). Although SNPs significantly affect the regulatory effects of CDS- and 5′UTR-located miRNA target sites, a systematic study on their influence is lacking.

To achieve this goal, we developed the MtiBase database to identify both CDS- and 5′UTR-located miRNA target sites and to uncover their regulatory effects on mRNA stability and translation. MtiBase provides comprehensive annotation of the miRNA–target interaction maps from multiple computational and experimental data. More importantly, the regulatory effects of miRNAs were evaluated using large amounts of high-throughput data, including 222 gene expression profiles, 28 RPF and six pSILAC datasets. Furthermore, the effects of SNPs on the functions of CDS- and 5′UTR-located miRNA target sites were systematically investigated.

## Methods

**Figure 1.**
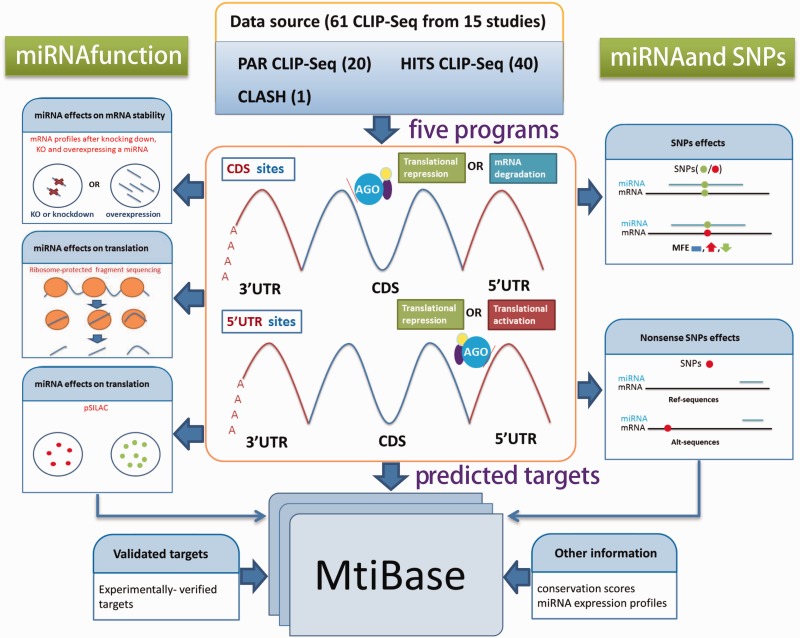
Systematic overview of MtiBase core framework. The results generated by MtiBase are stored in a MySQL database and displayed on the web page.

The analytical workflow consisted of three sections: CDS- and 5′UTR-located miRNA target prediction, functional data analysis and SNP-related target identification (Figure 1).

### CDS- and 5′UTR-located miRNA target site prediction

To identify the Ago-binding regions, 61 Ago CLIP-Seq (HITS-CLIP, PAR-CLIP and CLASH) datasets from human and mouse were downloaded from the National Center for Biotechnology Information Gene Expression Omnibus (NCBI GEO) (25) or starBase (13, 26) (details in Supplementary materials). Among the 61 datasets, the Ago-binding regions for 45 datasets were recognized by starBase; therefore, we totally followed their results. For the remaining 16 datasets that have not been annotated, we applied similar methods as that adopted in starBase to identify the Ago-binding regions. In brief, reads were first aligned to the reference genome (hg19 for human, mm9 for mouse) using Bowtie program (version 0.12.9) (27) with options: -v 2 -m 1 -best -strata. Reads aligned to multiple equivalent hits to the genome were discarded, and then the overlapping reads were gathered into clusters after removing the duplicate reads. Clusters with a minimum length of 20 nt and containing at least five reads were referred to as Ago-binding regions.

The miRNA target sites were predicted by five commonly used programs, including miRanda (28), RNA22 (29), miRWalk (30), microT-CDS (31) and STarMir (32). The results of RNA22, miRWalk, microT-CDS and STarMir were obtained from the respective websites. The results of miRanda were predicted using the mature miRNA sequences downloaded from miRBase (Release 21) (33) and the reference sequences of CDS and 5′UTR downloaded from BioMart of Ensembl (34). The hybridizing sequences of the target sites were then mapped to the reference genome to obtain their genomic coordinates using Bowtie. Finally, the CDS- and 5′UTR-located miRNA target sites overlapping the Ago-binding regions designated putative miRNA target sites. To compile the reference sequences, the mRNA sequences of protein-coding genes were downloaded from Ensembl, and sequences with complete coding regions were reserved. If a gene had several isoforms, the longest isoform was used to represent it. Then this set of sequences from unique genes was used as our reference sequence.

For species conservation analysis, PhastCons conservation scores (35) were downloaded from the University of California Santa Cruz bioinformatics websites (UCSC) (36). The mean conservation scores of the seed pairing regions were then calculated. The miRNA expression profiles across different tissues and cell lines were obtained from the Supplementary materials of a previous study and the corresponding CLIP-Seq experiments (37). The experimentally verified CDS- and 5′UTR-located target sites were curated from published studies.

### Functional data analysis

To assess the impacts of miRNAs on mRNA stability, we used 222 gene expression profiles (details in Supplementary materials) in response to miRNA overexpression, knockdown or knockout. The normalized data of microarray-detected gene expression profiles were downloaded from GEO of NCBI. Then the normalized probe intensities were extracted using the GEOquery package (38) of R software (www.R-project.org), and the log_2_ fold changes of each probe were calculated. If a gene had multiple probes, the mean fold changes of each probe were used to represent it. The RNA-seq quantified data of expression profile data were downloaded from the NCBI sequence read archive. Afterwards, reads were mapped to the reference genome using Bowtie. Reads aligned to multiple equivalent hits to the genome were discarded. To capture the reads spanning splice junctions, reads that did not align to the genome were mapped to the reference transcripts. The reads per kilobase per million reads method (39) was applied to quantify gene expression using the DEGseq package (40) in R and the log_2_ fold change of each gene was calculated.

Data from the RPF and pSILAC experiments were used to evaluate the effects of miRNAs on translational efficiency and protein synthesis, respectively (details in Supplementary materials). The processed RPF data and the corresponding mRNA profiles were downloaded from GEO. The log_2_ fold changes of the RPF data minus those of gene expression profiles to evaluate the changes of translational efficiency. The log_2_ fold changes of the pSILAC data were downloaded from the pSILAC database (20).

### SNP-related target identification

Information on human and mouse SNPs was downloaded from dbSNP of NCBI. The genomic coordinates of the SNPs were converted to those of the reference genome (hg19 or mm9) using the LiftOver utility in UCSC (36). To link the SNPs to human traits and diseases, we obtained genome-wide association studies (GWAS) data from the National Human Genome Research Institute (NHGRI GWAS Catalog) (41) and used it to annotate the SNPs. Then, the coordinates of the CDS- and 5′UTR-located target sites were compared with those of the SNPs to identify the target sites intersecting with SNPs. Then the hybridizing sequences of the overlapped target sites were extracted from the reference genome and converted to RNA sequences, and termed Ref-sequences. Subsequently, the corresponding allele was converted to an alternative allele in the Ref-sequence, i.e. Alt-sequence. The minimum free energy (MFE) of the Ref-sequence and Alt-sequence with the miRNAs was calculated using RNAhybrid (42). Nonsense SNPs in our reference transcripts were identified and the downstream portion of the CDS was redefined as an additional part of the 3′UTR. Gene ontology (GO) enrichment analysis of the genes with nonsense SNPs was performed using PANTHER (43). Lastly, CDS-located miRNA target sites within the additional part of the 3′UTR were identified.

## Results

### CDS- and 5′UTR-located miRNA target site identification and annotation

By analysing the 61 Ago CLIP-Seq datasets, we identified ∼260 000 and 34 000 Ago-binding regions within the CDS and 5′UTR regions, respectively (Table 1). Using the Ago-binding regions to limit the search space, we discovered about 4 400 000 CDS-located and 470 000 5′UTR-located miRNA target sites overlapping these regions (Table 1). The target sites involved a total of 15 546 genes and 4420 miRNAs (Supplementary Table S1). Analysis of the CLIP-Seq number of the targets revealed that the target numbers decreased significantly as the experimental support increased (Supplementary Figure S1). This indicates that targets predicted by more CLIP-Seq experiments may be more credible and that different prediction programs may generate a proportion of false positive targets.

**Table 1.** Data statistics in MtiBase

Species	Library	5′UTR cluster	CDS cluster	5′UTR site	CDS site
Human	56	33 861	256 544	467 692	4 272 636
Mouse	5	423	6175	1993	116 653

Data statistics are for library (CLIP-Seq), 5′UTR cluster (clusters of CLIP-Seq within 5′UTR), CDS cluster, 5′UTR site (miRNA target sites within 5′UTR), and CDS site.

To annotate the miRNA target sites, we integrated the data of the conservation scores and of the miRNA expression profiles. We proved that the CDS- and 5′UTR-located miRNA binding sites have been proved evolutionarily conserved (15–17). Analysis of the conservation of our predicted targets revealed that their conservation scores increased with experimental support (Supplementary Figure S2). In addition, miRNA has temporal and tissue-specific expression (37). Therefore, we integrated miRNA expression profiles across various tissues and cell lines to help further reduce the false positive rates and prioritize the search results. The tissue miRNA expression profiles revealed that the number of ∼16% of miRNAs was >1 RPM (Supplementary Figure S3). The remaining miRNAs showed extremely low expression or no expression (Supplementary Figure S3).

To learn about the experimentally validated CDS- and 5′UTR-located miRNA target sites, we recaptured them from published studies. In total, MtiBase curated 27 CDS-located and eight 5′UTR-located miRNA target sites with genomic coordinates (Supplementary Table S2). These sites were verified by polymerase chain reaction, western blot or reporter assays. Furthermore, we integrated details about the interactions, such as binding condition, genomic coordinates and validated methods (Supplementary Figure S4). Subsequently, we searched the validated target sites from our predicted targets, and found that MtiBase recaptured nearly 10 validated sites (Supplementary Table S3). For example, it recaptured the interaction between miR-183 and beta-transducin repeat containing (*BTRC*) that could trigger the degradation of *BTRC* mRNA (44).

### Regulatory effects quantification

To assess the regulatory effects of miRNAs, we integrated multiple high-throughput experimental data, including 222 gene expression profiles, 28 RPF and six pSILAC datasets (Table 2). These experimental data involved 84 human miRNAs and 30 mouse miRNAs. The fold changes of these data can help reduce the false positive rates according to the experimental treatment. For example, the results of several gene expression profiles after a miRNA had been overexpressed showed that the expression levels of most predicted CDS-located targets would be down regulated after overexpressing a miRNA (Supplementary Figure S5), indicating that most putative targets may be credible. The experimental data can be used to not only reduce false positive rates, but also assess the effects of miRNAs on mRNA stability, transcription efficiency or protein synthesis.

**Table 2.** Experimental data used to quantify miRNA regulatory effects

Species	mRNA	RPF	pSILAC
Human	159	6	6
Mouse	63	22	0

RPF, ribosome-protected fragment sequencing; pSILAC, pulsed stable isotope labeling with amino acids in culture.

### MiRNA functions under the influence of SNPs

SNPs influence the regulatory effects of miRNAs by disrupting miRNA–target interactions (23). To identify SNP-disrupted miRNA–target interactions, we first identified ∼280 000 and 28 000 SNPs overlapping the mRNA CDS and 5′UTR regions, respectively (Supplementary Table S4). Comparison of the genomic coordinates of the CDS- and 5′UTR-located miRNA target sites with those of the SNPs revealed 2 900 000 CDS-located and 28 000 5′UTR-located miRNA target sites overlapping these SNPs (Supplementary Table S4). Previous studies have proven that SNPs may affect the binding affinity between miRNAs and their targets to enhance or weaken the effects of miRNAs on mRNA stability (23). Therefore, we calculated the MFE changes using the Ref-sequences and Alt-sequences with the miRNAs to evaluate the changes of binding affinity under SNP influence. The MFE of most interactions increased after the corresponding allele was converted to the alternative allele in the hybridizing sequences (Supplementary Figure S6), which indicates that most SNPs might weaken the effects of miRNAs on mRNA stability. On the other hand, these SNPs could also enhance the binding affinity of miRNA–target interactions, but these interactions merely comprised ∼25% of the total interactions.

Moreover, the CDS-located target sites could act as a surveillance system to induce the degradation of nonsense mRNAs (24). Thus, we first identified nearly 4900 and 10 nonsense SNPs in the CDS of human and mouse, respectively (Supplementary Table S5). These SNPs involved 4862 human and eight mouse protein coding genes (Supplementary Table S5). Analysis of the function of these genes determined that >50% of them were metabolism-related, indicating the important roles of this surveillance system in metabolism (Supplementary Table S6). Then, we identified ∼1 100 000 human and 400 mouse CDS-located target sites in the additional 3′UTR that may participate in the surveillance system (Supplementary Table S5).

### Database introduction

**Figure 2.**
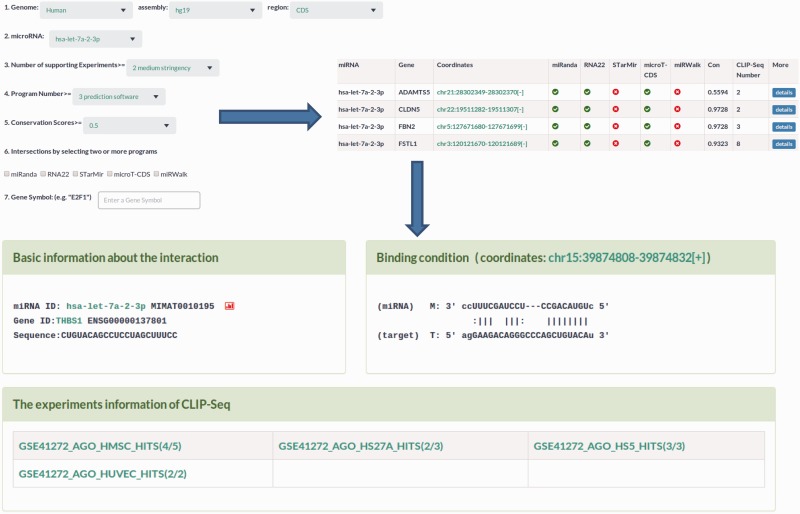
Sample output image for predicted miRNA target sites. MtiBase provides various options for users to optimize their search results. Additional information on the miRNA target sites could be obtained by clicking the ‘details’ button.

**Figure 3.**
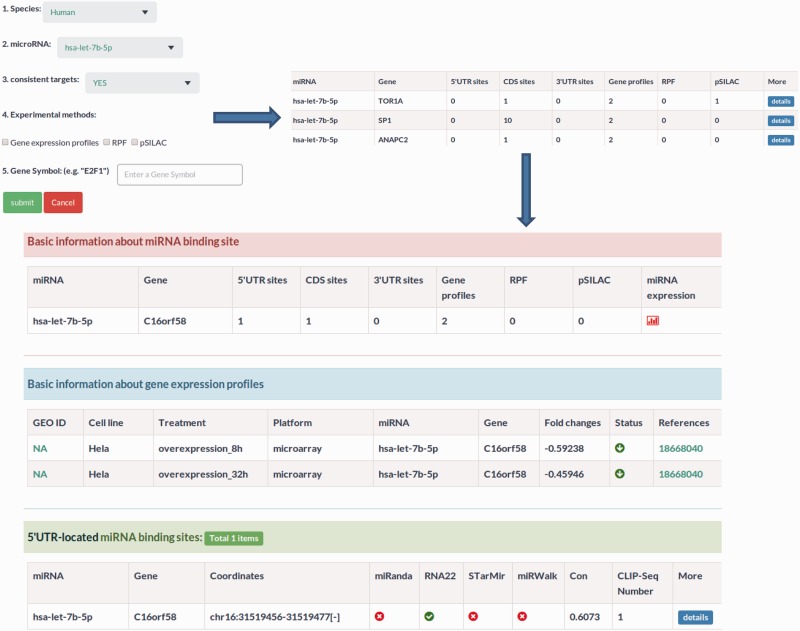
Sample output image for miRNA function section. Apart from miRNA–target regulatory relationships, MtiBase also integrates the experimental data on the relationships to assess the effects of miRNAs on transcription and translation.

MtiBase has a user-friendly interface and various options to facilitate investigation of CDS- and 5′UTR-located miRNA target sites. The database consists of four main sections: putative target section, miRNA effect section, SNPs section and validated target section. The putative target section offers the predicted CDS- and 5′UTR-located miRNA target sites as well as their details (Figure 2). This section provided three options to optimize search results, including prediction programs, the number of CLIP-Seq and conservation scores (Figure 2). The miRNA function section contains the fold changes of mRNA expression levels, translational efficiency and protein synthesis in response to miRNA overexpression, knockdown or knockout (Figure 3). In this section, users can learn about the regulatory effects of miRNAs on mRNA stability and translation by choosing the corresponding experimental approaches, such as gene expression profiles for mRNA stability or pSILAC for protein synthesis. Besides, the section provides more details about the interactions, such as the miRNA expression profiles (Supplementary Figure S7). The SNP section lists the CDS- and 5′UTR-located miRNA target sites under the influence of SNPs (Supplementary Figure S8). The validated target section contains experimentally validated CDS- and 5′UTR-located miRNA targets. Additional information about the validated interactions can be obtained by clicking the ‘details’ button (Supplementary Figure S4).

## Discussion

In this study, we integrated multiple experimental and computational data to identify CDS- and 5′UTR-located miRNA targets and to assess their regulatory effects on mRNA stability and translation. We also systematically investigated the influence of SNPs on the regulatory effects of these sites. We identified an increasing number of CDS- and 5′UTR-located miRNA target sites, which may reveal the complexity and universality of miRNA regulation (Table 1).

To minimize the rates of false positive target prediction, MtiBase integrated a great quantity of data in each procedure. In target prediction, it incorporated the miRNA target sites generated by five different prediction programs and the data from 61 Ago CLIP-Seq datasets. Target sites predicted by more programs and that have more experimental support, i.e. CLIP-Seq, may be more credible. In target annotation, conservation scores could be applied to filter the less conserved targets, since functional targets are more likely to be conserved between different species. In regulatory effects quantification, the experimental data from 222 expression profiles, 28 RPF and six pSILAC datasets were used. The fold changes of these data in response to miRNA overexpression, knockdown or knockout can help further reduce false positive rates.

Previous studies have proven that SNPs can significantly affect the regulatory effects of CDS- and 5′UTR-located miRNA target sites (23, 24). Our results reveal that the MFE of most miRNA–target interactions increased after converting the corresponding allele to an alternative allele, which may reflect that SNPs mainly weaken miRNA regulatory effects on mediating mRNA degradation and translation by reducing their binding affinity. Besides, the CDS-located target sites can act as a surveillance system to induce the degradation of nonsense mRNAs (24). Analysis of the genes with nonsense SNPs revealed that most of the enriched GO terms were metabolism-related (Supplementary Table S6), which may reflect the fact that cells expend significant resources on surveillance of the metabolic system.

Compared with other miRNA target-related databases, which include miRecord (45) and TarBase (46), which merely contain experimentally supported targets, the distinctive features of the MtiBase database are as follows: (i) It used the data from 61 Ago CLIP-Seq datasets to reduce false positive target prediction rates. (ii) It calculated the conservation scores of target sites and integrated miRNA expression profiles across different tissues and cell lines to help decrease the false positive rate of miRNA target sites. (iii) In addition to identifying CDS- and 5′UTR-located target sites, it enabled assessment of the regulatory effects of miRNAs on mRNA stability and translation by integrating large amounts of experimental data, including 222 gene expression profiles, 28 RPF and six pSILAC. These experimental data can also be used to further reduce the rates of false positive target prediction. (iv) It could systematically investigate the influence of SNPs on the regulatory effects of CDS- and 5′UTR-located miRNA target sites. (v) It recaptured experimentally validated CDS- and 5′UTR-located miRNA target sites and their details from the published literature.

In summary, MtiBase integrates multiple experimental and computational data to decode the functional CDS- and 5′UTR-located miRNA target sites. We hope that MtiBase could comprehensively improve our understanding of the important roles of miRNAs in the regulation of gene expression and the processes of physiology and pathology.

## Supplementary Data

Supplementary data are available at *Database* Online.

## Funding

This work was supported by project grants from the National Basic Research Program of China (2011CB811305); the National Natural Science Foundation of China (81230073, 31370791, 91440110, 91442205); Project of Science and Technology New Star in ZhuJiang Guangzhou city (2012J2200025). Funding for open access charge: National Natural Science Foundation of China (81230073 and 91442205).

*Conflict of interest*. None declared.

## Supplementary Material

Supplementary Data
