# Supplementary material for: MtiBase: a database for decoding microRNA target sites located within CDS and 5′UTR regions from CLIP-Seq and expression profile datasets
Source: Database (Oxford). 2015 Oct 21;2015:bav102. doi: 10.1093/database/bav102 (PMC4614282; doi:10.1093/database/bav102)
Supplement: Supplementary Data [file supp_2015_bav102_index.html]

Supplementary Data 

# MtiBase: a database for decoding microRNA target sites located within CDS and 5′UTR regions from CLIP-Seq and expression profile datasets

## Supplementary Data

files

- Supplementary Data - doc file
