# Supplementary material for: MtiBase: a database for decoding microRNA target sites located within CDS and 5′UTR regions from CLIP-Seq and expression profile datasets
Source: Database (Oxford). 2015 Oct 21;2015:bav102. doi: 10.1093/database/bav102 (PMC4614282; doi:10.1093/database/bav102)
Supplement: Supplementary Data [file supp_bav102_2015_8_27_MtiBase_supplement.doc]

| Supplementary Material  **MtiBase: a database for decoding microRNA target sites located within CDS and 5′UTR regions from CLIP-Seq and expression profile datasets**  Zhi-Wei Guo^1^, Chen Xie^2^, Jian-Rong Yang^3^, Jun-Hao Li^1^, Jian-Hua Yang^1,*^ and Limin Zheng^1, 4,*^  ^1^Key Laboratory of Gene Engineering of the Ministry of Education, State Key Laboratory of Biocontrol, Sun Yat-sen University, Guangzhou, P. R. China  ^2^Key Laboratory of Liver Disease of Guangdong Province, The Third Affiliated Hospital of Sun Yat-sen University, Guangzhou, P. R. China  ^3^Department of Ecology and Evolutionary Biology, University of Michigan, Ann Arbor, MI, USA.  ^4^Collaborative Innovation Center for Cancer Medicine, Sun Yat-sen University Cancer Center, State Key Laboratory of Oncology in South China, Guangzhou, P. R. China |
| --- |

**Supplementary Tables, Figures and Information**

| Supplementary Table | |
| --- | --- |
| Supplementary Table S1 | Gene and miRNA derived from miRNA–target interactions. |
| Supplementary Table S2 | Experimentally validated CDS- and 5′UTR-located miRNA target sites |
| Supplementary Table S3 | Experimentally validated miRNA target sites recaptured by MtiBase. |
| Supplementary Table S4 | Number of the SNPs and SNP-overlapped miRNA target sites. |
| Supplementary Table S5 | Number of nonsense SNPs and related target sites. |
| Supplementary Table S6 | Enriched GO analysis of the genes with nonsense SNPs. |
| Supplementary Figure | |
| Supplementary Figure S1 | Nnumber of miRNA target sites with different CLIP-Seq numbers. |
| Supplementary Figure S2 | Correlation between conservation scores and CLIP-Seq number of miRNA target sites. |
| Supplementary Figure S3 | Distribution of miRNA expression across different tissues. |
| Supplementary Figure S4 | Sample output image for validated CDS-located miRNA target sites |
| Supplementary Figure S5 | Examples of fold changes of gene expression in response to miRNA overexpression. |
| Supplementary Figure S6 | MFE changes under the influence of SNPs. |
| Supplementary Figure S7 | Sample output image for miRNA expression levels across different tissues. |
| Supplementary Figure S8 | Sample output image for SNP-related miRNA target sites. |
| Data Information |  |
| Expression profiles | Information about gene expression profiles used in MtiBase |
| RPF | Information about RPF used in MtiBase |
| pSILAC | Information about pSILAC used in MtiBase |
| Ago CLIP-Seq | Information about Ago CLIP-Seq used in MtiBase |

**SUPPLEMENTARY TABLES**

**Table S1.** Gene and miRNA derived from miRNA–target interactions.

| **Species** | **5′UTR Gene** | **CDS Gene** | **5′UTR miRNA** | **CDS miRNA** | **miRNA** | **Gene** |
| --- | --- | --- | --- | --- | --- | --- |
| Human | 6,934 | 12,432 | 2,452 | 2,569 | 2,579 | 12,795 |
| Mouse | 112 | 2,751 | 722 | 1,834 | 1,841 | 2,751 |

The gene means the number of genes involving the miRNA–target interactions. The miRNA represents the miRNAs derived from the interactions. Gene and miRNA means the number of genes and miRNAs involving the interactions.

**Table S2.** Experimentally validated CDS- and 5′UTR-located miRNA target sites.

| miRNA | Gene | Region | Genomic coordinates (hg19 or mm9) |
| --- | --- | --- | --- |
| hsa-miR-24-3p | HNF4A | CDS | chr20:43043239-43043260 [+] |
| hsa-miR-24-3p | HNF4A | CDS | chr20:43047092-43047113 [+] |
| hsa-miR-24-3p | HNF4A | CDS | chr20:43048390-43048413 [+] |
| hsa-miR-24-3p | HNF4A | CDS | chr20:43052697-43052722 [+] |
| hsa-miR-24-3p | HNF4A | CDS | chr20:43058193-43058216 [+] |
| hsa-miR-148a-3p | DNMT3B | CDS | chr20:31394095-31394124 [+] |
| hsa-let-7a-5p | DICER1 | CDS | chr14:95570085-95570106 [-] |
| hsa-let-7a-5p | DICER1 | CDS | chr14:95569977-95569994 [-] |
| hsa-let-7a-5p | DICER1 | CDS | chr14:95560443-95560467 [-] |
| hsa-miR-138-5p | FOSL1 | CDS | chr11:65660611-65660637 [-] |
| hsa-miR-138-5p | FOSL1 | CDS | chr11:65660601-65660624 [-] |
| hsa-miR-138-5p | FOSL1 | CDS | chr11:65660591-65660612 [-] |
| hsa-miR-24-3p | FAF1 | CDS | chr1:51032800-51032818 [-] |
| hsa-miR-24-3p | FAF1 | CDS | chr1:50941310-50941330 [-] |
| hsa-miR-107 | INSIG1 | CDS | chr7:155090171-155090193 [+] |
| hsa-miR-107 | INSIG1 | CDS | chr7:155090287-155090312 [+] |
| hsa-miR-30b-5p | RAB18 | CDS | chr10:27822656-27822678 [+] |
| hsa-miR-30c-5p | RAB18 | CDS | chr10:27822662-27822678 [+] |
| hsa-miR-181a-5p | ZNF37A | CDS | chr10:38407291-38407319 [+] |
| hsa-miR-181a-5p | ZNF37A | CDS | chr10:38407372-38407403 [+] |
| hsa-miR-181a-5p | ZNF37A | CDS | chr10:38407625-38407655 [+] |
| hsa-miR-185 | MZB1 | CDS | chr5:138723603-138723730 [-] |
| hsa-miR-183-5p | BTRC | CDS | chr10:103292736-103292758 [+] |
| hsa-miR-138-5p | FOSL1 | 5′UTR | chr11:65667817-65667844 [-] |
| hsa-miR-34a-5p | AXIN2 | 5′UTR | chr17:63557692-63557715 [-] |
| hsa-miR-34a-5p | WNT1 | 5′UTR | chr12:49375674-49375693 [+] |
| hsa-miR-605-5p | SEC24D | 5′UTR | chr4:119757172-119757197 [-] |
| hsa-miR-103a-3p | GPRC5A | 5′UTR | chr12:13044045-13044067 [+] |
| mmu-miR-2861 | HDAC5 | CDS | chr11:102065921-102065939 [-] |
| mmu-miR-296-5p | Nanog | CDS | chr6:122661639-122661659 [+] |
| mmu-miR-470-5p | Nanog | CDS | chr6:122661671-122661691 [+] |
| mmu-miR-134-5p | Sox2 | CDS | chr3:34549963-34549984 [+] |
| mmu-miR-10a-5p | RPS16 | 5′UTR | chr7:28350755-28350777 [+] |
| mmu-miR-10a-5p | RPL13A | 5′UTR | chr7:45128696-45128721 [-] |
| mmu-miR-10a-5p | RPS20 | 5′UTR | chr4:3762717-3762752 [-] |

This table lists the validated miRNA target sites with genomic coordinates.

**Table S3.** Experimentally validated miRNA target sites recaptured by MtiBase.

| miRNA | Gene | Region | Genomic coordinates |
| --- | --- | --- | --- |
| hsa-let-7a-5p | DICER1 | CDS | chr14:95570085-95570106 [-] |
| hsa-let-7a-5p | DICER1 | CDS | chr14:95569977-95569994 [-] |
| hsa-let-7a-5p | DICER1 | CDS | chr14:95560443-95560467 [-] |
| hsa-miR-138-5p | FOSL1 | CDS | chr11:65667817-65667844 [-] |
| hsa-miR-138-5p | FOSL1 | CDS | chr11:65660611-65660637 [-] |
| hsa-miR-138-5p | FOSL1 | CDS | chr11:65660601-65660624 [-] |
| hsa-miR-24-3p | FAF1 | CDS | chr1:50941310-50941330 [-] |
| hsa-miR-107 | INSIG1 | CDS | chr7:155090287-155090312 [+] |
| hsa-miR-183-5p | BTRC | CDS | chr10:103292736-103292758 [+] |

**Table S4.** Number of the SNPs and SNP-overlapped miRNA target sites.

| Species | 5′UTR SNPs | CDS SNPs | 5′UTR sites | CDS sites |
| --- | --- | --- | --- | --- |
| Human | 27,990 | 274,779 | 27,990 | 2,856,395 |
| Mouse | 77 | 3,515 | 243 | 20,355 |

The 5′UTR and CDS SNPs represent the SNPs overlap mRNA 5′UTR and CDS, respectively. The sites mean the miRNA target sites overlapping with these SNPs.

**Table S5.** Number of nonsense SNPs and related target sites.

| Species | Nonsense SNPs | Related gene | Target sites |
| --- | --- | --- | --- |
| Human | 4,862 | 4,862 | 1,057,448 |
| Mouse | 8 | 8 | 369 |

Target sites represent the CDS-located target sites within the additional part of 3′UTR formed by nonsense SNPs.

**Table S6.** Enriched GO analysis of the genes with nonsense SNPs.

| No. | GO Biological Process | P value |
| --- | --- | --- |
| 1 | metabolic process | 2.18e-60 |
| 2 | Primary metabolic process | 2.28E-42 |
| 3 | nucleobase-containing compound metabolic process | 9.27E-21 |
| 4 | DNA repair | 2.19E-16 |
| 5 | DNA metabolic process | 1.12E-12 |
| 6 | protein metabolic process | 9.94E-11 |
| 7 | cellular component organization or biogenesis | 1.56E-07 |
| 8 | protein transport | 3.35E-07 |
| 9 | intracellular protein transport | 5.76E-07 |
| 10 | RNA metabolic process | 6.10E-07 |

The P-values were calculated by PANTHER.

**SUPPLEMENTARY FIGURES**

**
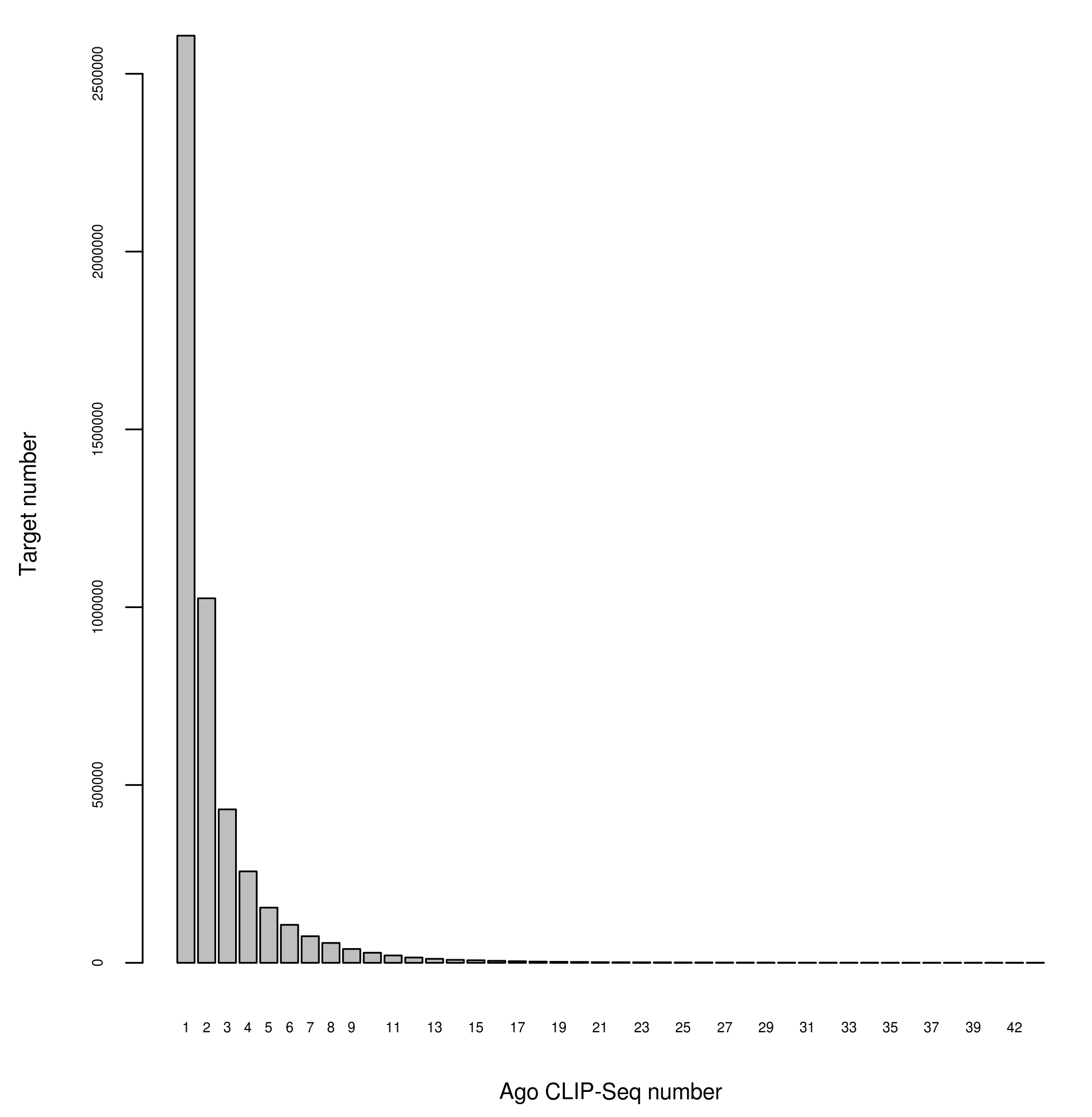
**

**Figure S1. Number of miRNA target sites with different CLIP-Seq numbers.** Ago CLIP-Seq numbers represent the numbers of CLIP-Seq experiments where the Ago-binding regions overlap the miRNA target sites.


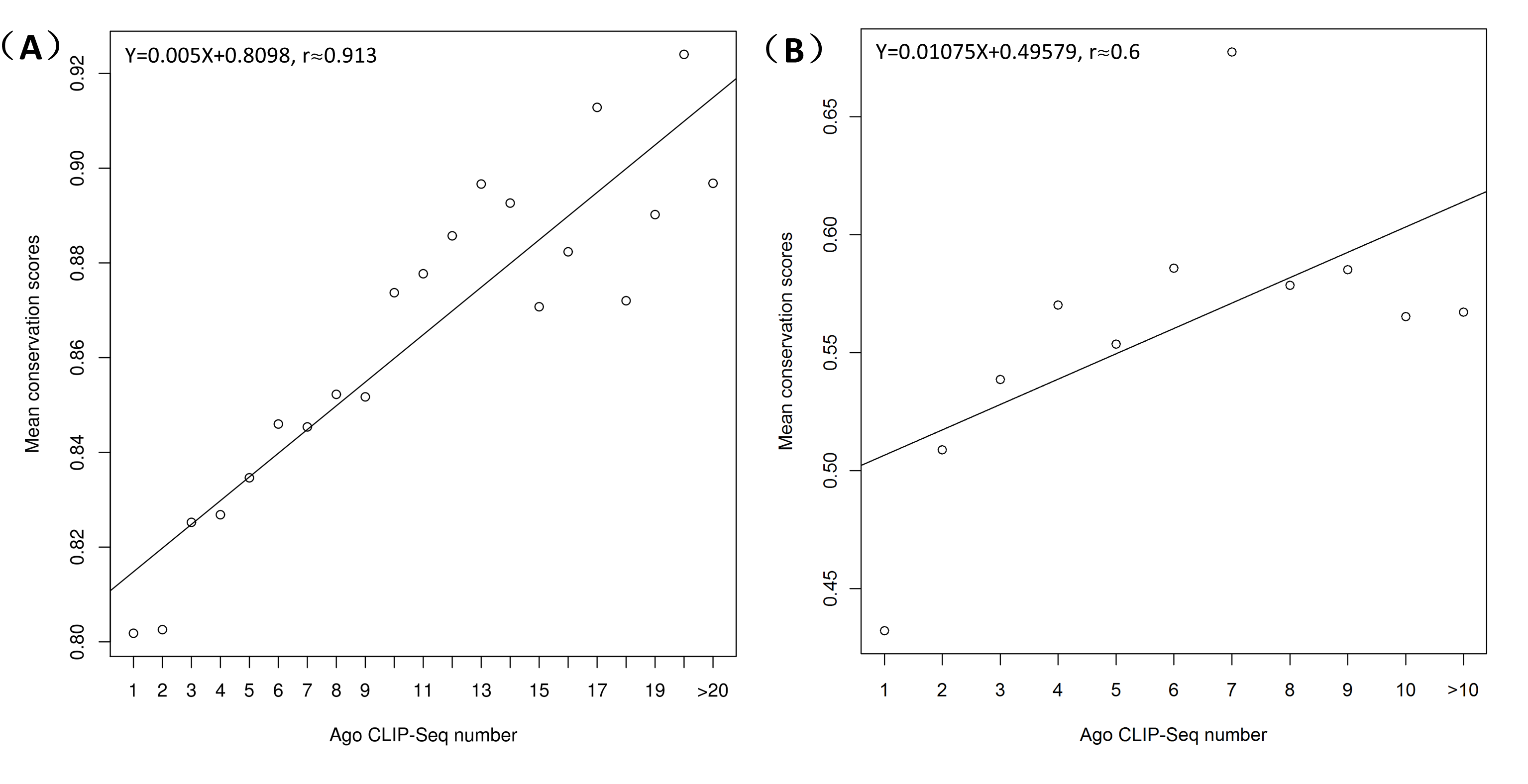


**Figure S2. Correlation between conservation scores and CLIP-Seq number of miRNA target sites. (A) CDS-located miRNA target sites; (B) 5′UTR-located miRNA target sites.** The P-values of the Pearson correlation of the CDS- and 5′UTR-located target sites were approximately 8.18e-09 and 0.05, respectively.


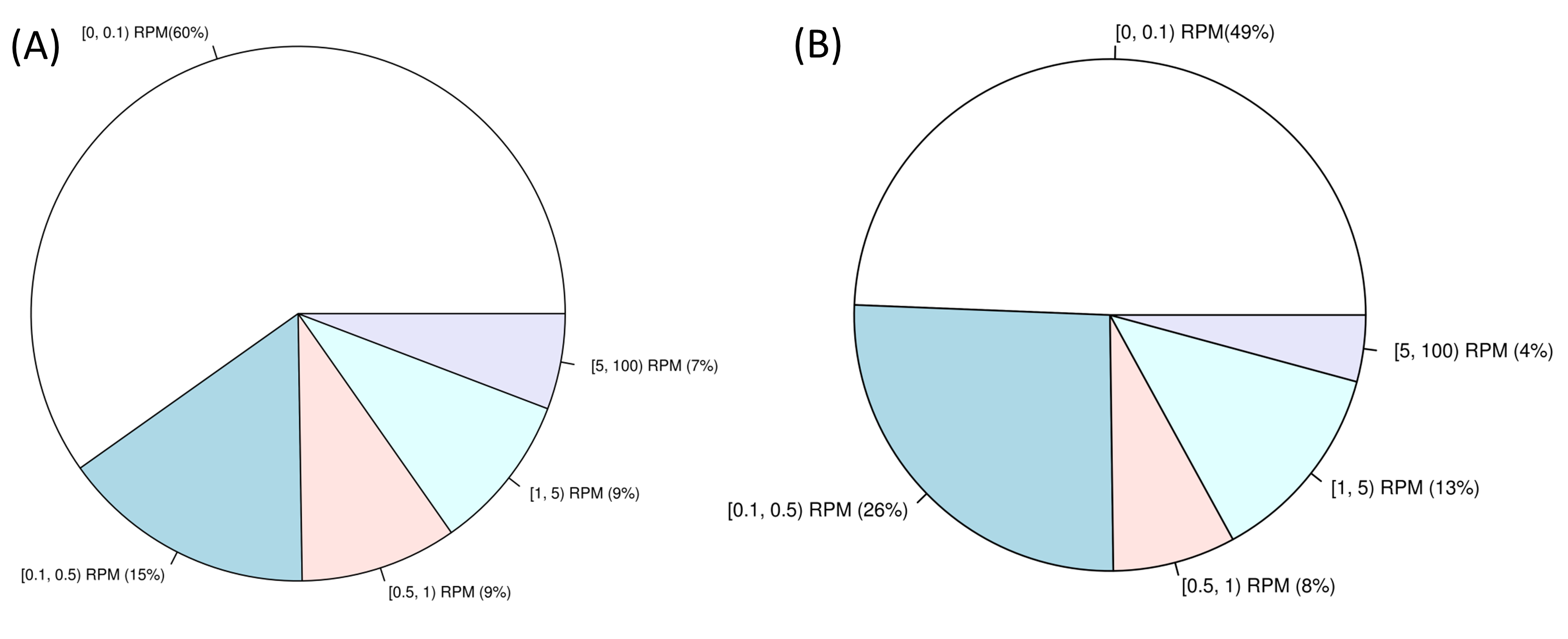


**Figure S3. Distribution of miRNA expression across different tissues. (A) Human; (B) mouse.**


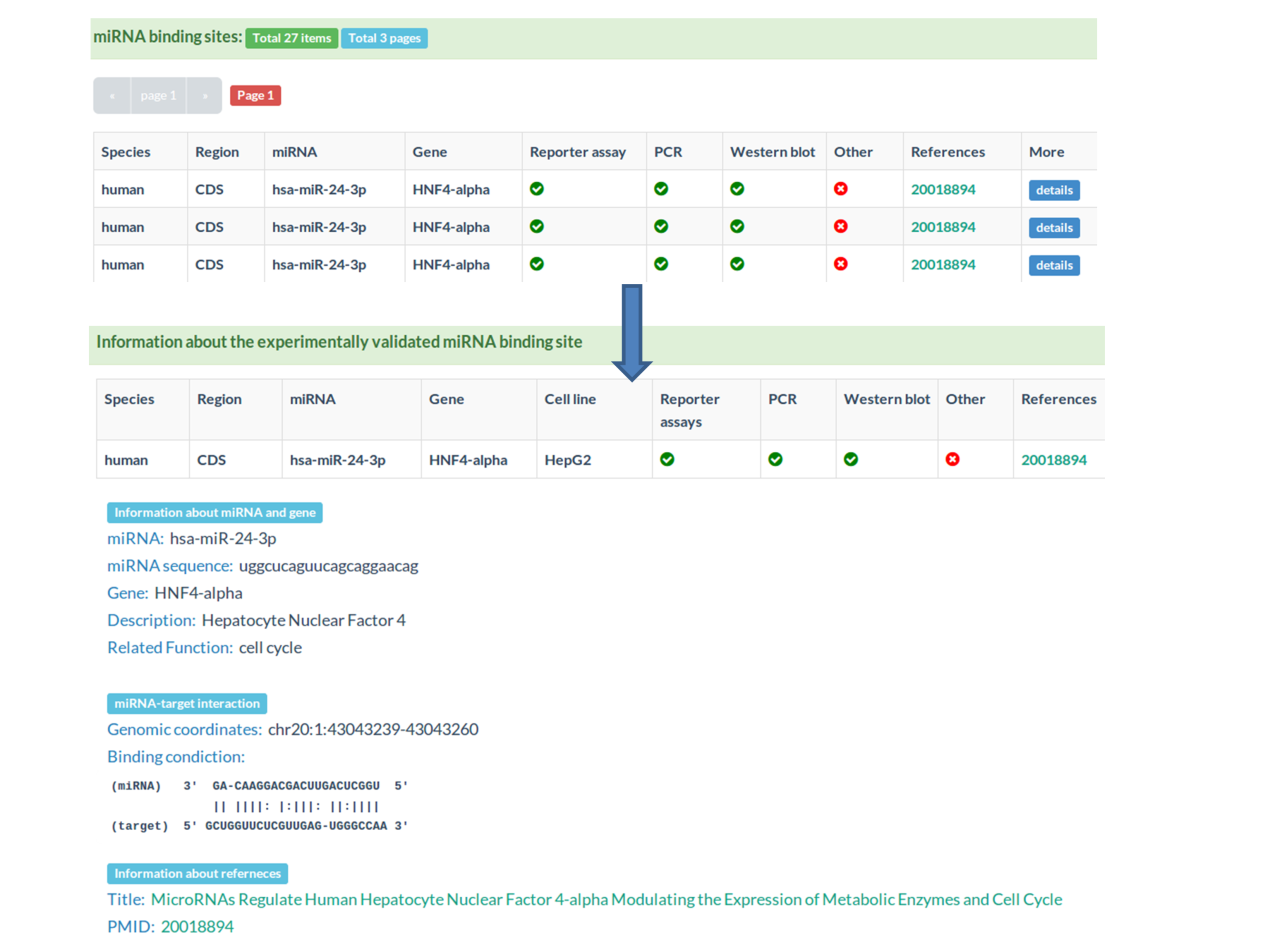


**Figure S4. Sample output image for validated CDS-located miRNA target sites.**


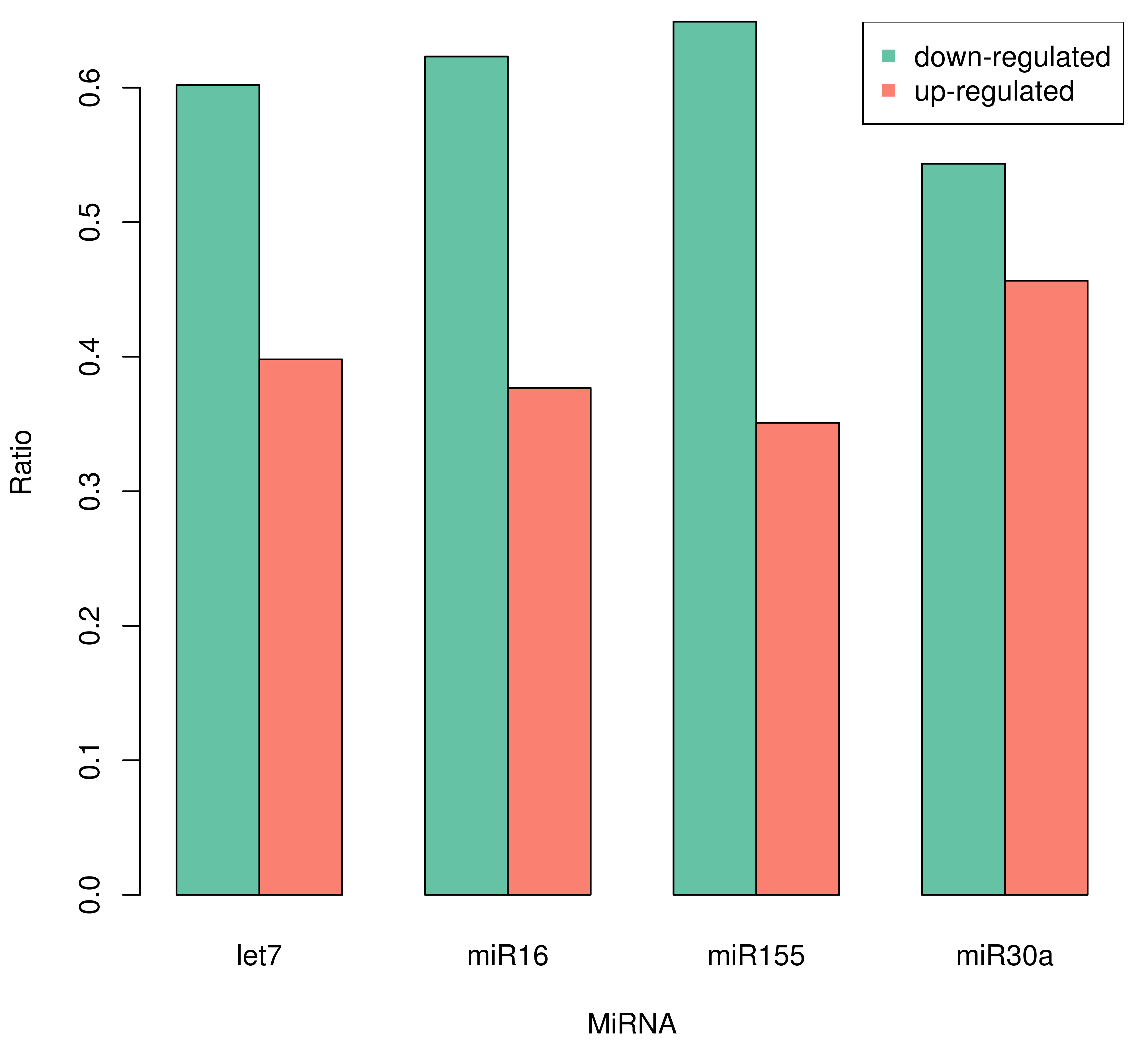


**Figure S5. Examples of fold changes of gene expression in response to miRNA overexpression.** Four samples are shown: let-7, miR-16, miR-155, and miR-30a.


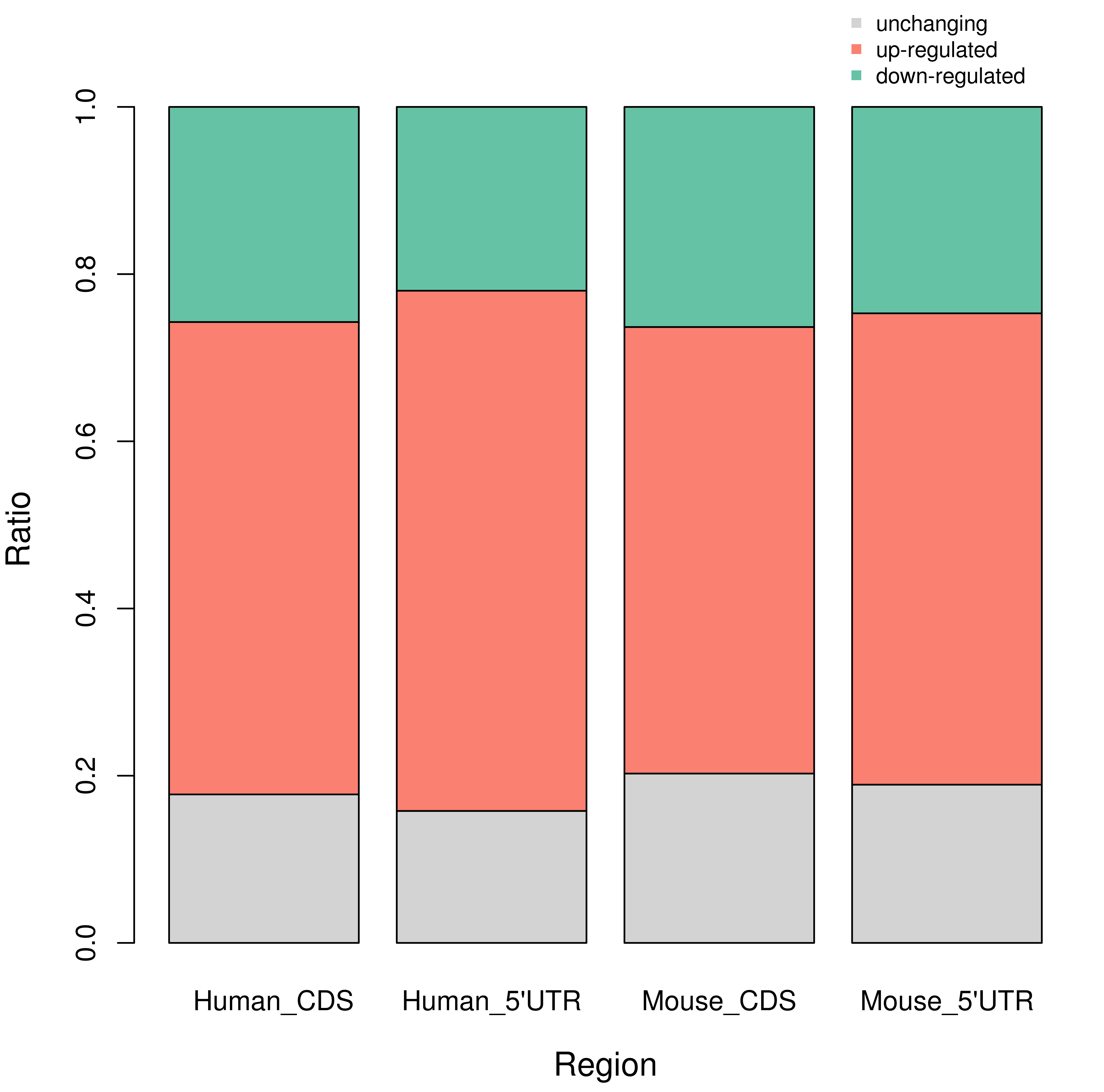


**Figure S6. MFE changes under the influence of SNPs.** The MFE reflects the binding affinity between miRNAs and their targets. A larger MFE indicates weaker binding affinity. Our results showed that the MFE of most miRNA–target interactions was increased and that only a small proportion of interactions was decreased.


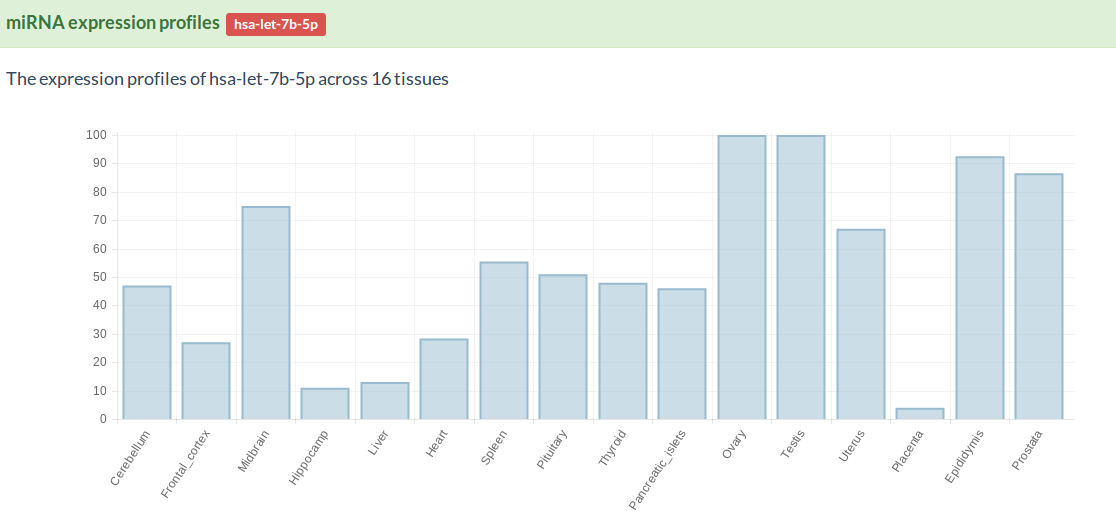


**Figure S7. Sample output image for miRNA expression levels across different tissues.**


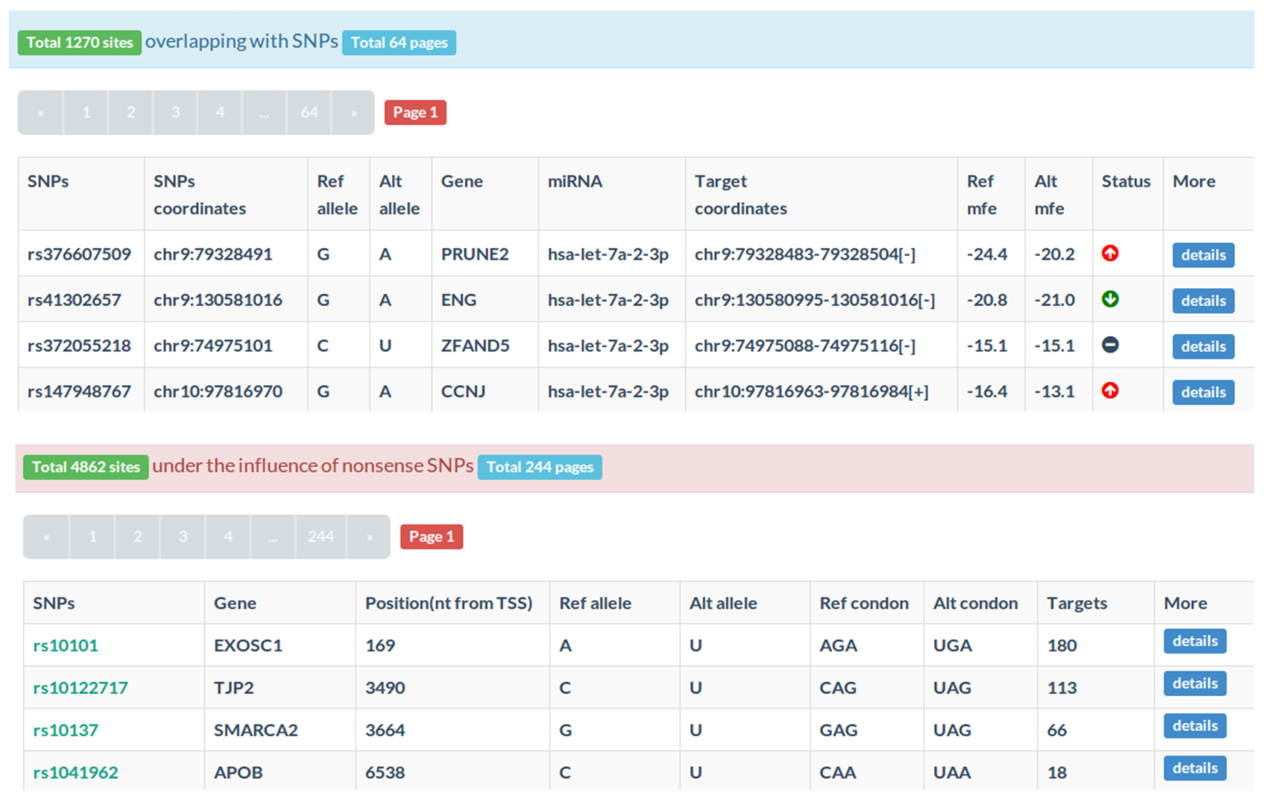


**Figure S8. Sample output image for SNP-related miRNA target sites.**

**Data information**

The information about the experimental data (gene expression profiles, RPF, pSILAC and Ago CLIP-Seq) integrated by MtiBase.

**Gene expression profiles integrated by MtiBase.**

| No. | miRNA | Species | Cell line | Treatment | Reference |
| --- | --- | --- | --- | --- | --- |
| 1 | hsa-miR-26a-5p | human | Namalwa | overexpression | Sandar, et al. 2008 |
| 2 | hsa-miR-26a-5p | human | Raji | overexpression | Sandar, et al. 2008 |
| 3 | hsa-miR-26a-5p | human | Ramos | overexpression | Sandar, et al. 2008 |
| 4 | hsa-miR-7-5p | human | HEK-293 | overexpression | Hausser, et al. 2009 |
| 5 | hsa-miR-124-3p | human | HEK-293 | overexpression | Hausser, et al. 2009 |
| 6 | hsa-miR-9-5p | human | MCF-7 | overexpression | Hsu, et al. 2009 |
| 7 | hsa-miR-9-3p | human | MCF-7 | overexpression | Hsu, et al. 2009 |
| 8 | hsa-miR-34a-5p | human | K562 | overexpression | Navarro, et al. 2009 |
| 9 | hsa-miR-210-3p | human | HUVEC | overexpression | Fasanaro, et al. 2009 |
| 10 | hsa-miR-210-3p | human | HUVEC | anti | Fasanaro, et al. 2009 |
| 11 | hsa-miR-130a-3p | human | LNCaP | overexpression | Boll, et al. 2013 |
| 12 | hsa-miR-205-5p | human | LNCaP | overexpression | Boll, et al. 2013 |
| 13 | hsa-miR-145-5p | human | DLD-1 | overexpression | Gregersen, et al. 2010 |
| 14 | hsa-miR-20a-5p | human | QK-knockdown Hs683 | overexpression | Chen, et al. 2012 |
| 15 | hsa-miR-221-3p | human | MCF7-FR(fulvestrant-resistant) | si | Rao, et al. 2011 |
| 16 | hsa-miR-222-3p | human | MCF7-FR(fulvestrant-resistant) | si | Rao, et al. 2011 |
| 17 | hsa-miR-212-3p | human | HEK-293 | overexpression | Hollander, et al. 2010 |
| 18 | hsa-miR-7-5p | human | HEY | overexpression | Shahab, et al. 2011 |
| 19 | hsa-miR-128-3p | human | HEY | overexpression | Shahab, et al. 2011 |
| 20 | hsa-miR-30d-5p | human | melanoma cell line 4L | overexpression | Gaziel-Sovran, et al. 2011 |
| 21 | hsa-miR-30d-5p | human | melanoma cell line 5B1 | overexpression | Gaziel-Sovran, et al. 2011 |
| 22 | hsa-miR-101-3p | human | MCF-7 | overexpression | Frankel, et al. 2011 |
| 23 | hsa-miR-206 | human | LNCaP | overexpression | Hudson, et al. 2012 |
| 24 | hsa-miR-27b-3p | human | LNCaP | overexpression | Hudson, et al. 2012 |
| 25 | hsa-miR-1-3p | human | LNCaP | overexpression | Hudson, et al. 2012 |
| 26 | hsa-miR-124-3p | human | PDGFRA amplified neurospheres | overexpression | Setty, et al. 2012 |
| 27 | hsa-miR-132-3p | human | PDGFRA amplified neurospheres | overexpression | Setty, et al. 2012 |
| 28 | hsa-miR-380-3p | human | PDGFRA amplified neurospheres | overexpression | Setty, et al. 2012 |
| 29 | hsa-miR-433-3p | human | PDGFRA amplified neurospheres | overexpression | Setty, et al. 2012 |
| 30 | hsa-miR-448 | human | PDGFRA amplified neurospheres | overexpression | Setty, et al. 2012 |
| 31 | hsa-miR-143-3p | human | DLD-1 | overexpression | Gregersen, et al. 2012 |
| 32 | hsa-miR-376a-5p | human | u87 | overexpression | Choudhury, et al. 2012 |
| 33 | hsa-miR-129-2-3p | human | hTERT-RPE1 | overexpression_72h | Cao, et al. 2012 |
| 34 | hsa-miR-106b-5p | human | LNCaP | overexpression | Hudson, et al. 2013 |
| 35 | hsa-miR-941 | human | HSF2 | overexpression | Hu, et al. 2012 |
| 36 | hsa-miR-941 | human | HEK | overexpression | Hu, et al. 2012 |
| 37 | hsa-miR-941 | human | HEK293T | overexpression | Hu, et al. 2012 |
| 38 | hsa-miR-23b-3p | human | fibroblast-like synoviocytes | overexpression | Zhu, et al. 2012 |
| 39 | hsa-miR-27a-3p | human | Huh7.5 | overexpression | Shirasaki, et al. 2013 |
| 40 | hsa-miR-27a-3p | human | Huh7.5 | anti | Shirasaki, et al. 2013 |
| 41 | hsa-miR-146a-5p | human | HT29 | overexpression | Hwang, et al. 2014 |
| 42 | hsa-miR-483-5p | human | MHH-ES-1 | overexpression | Liu, et al. 2013 |
| 43 | hsa-miR-483-3p | human | MHH-ES-1 | overexpression | Liu, et al. 2013 |
| 44 | hsa-miR-210-3p | human | AGS | overexpression | Kiga, et al. 2014 |
| 45 | hsa-miR-210-3p | human | MKN45 | overexpression | Kiga, et al. 2014 |
| 46 | hsa-miR-124-3p | human | HepG2 | overexpressionion_4h | [Wang. 2006](#_ENREF_70) |
| 47 | hsa-miR-124-3p | human | HepG2 | overexpressionion_8h | [Wang. 2006](#_ENREF_70) |
| 48 | hsa-miR-124-3p | human | HepG2 | overexpressionion_16h | [Wang. 2006](#_ENREF_70) |
| 49 | hsa-miR-124-3p | human | HepG2 | overexpressionion_24h | [Wang. 2006](#_ENREF_70) |
| 50 | hsa-miR-124-3p | human | HepG2 | overexpressionion_32h | [Wang. 2006](#_ENREF_70) |
| 51 | hsa-miR-124-3p | human | HepG2 | overexpressionion_72h | [Wang. 2006](#_ENREF_70) |
| 52 | hsa-miR-124-3p | human | HepG2 | overexpressionion_120h | [Wang. 2006](#_ENREF_70) |
| 53 | hsa-miR-335-5p | human | LM2 | overexpression | Tavazoie, et al. 2008 |
| 54 | hsa-miR-138-5p | human | Calu-3 | overexpression | Ramachandran, et al. 2012 |
| 55 | hsa-miR-126-3p | human | HUVEC | anti | Fish, et al. 2008 |
| 56 | hsa-miR-29a-3p | human | imr90 | overexpression | Cushing, et al. 2011 |
| 57 | hsa-miR-517a-3p | human | HEK293T | overexpression | Toffanin, et al. 2011 |
| 58 | hsa-miR-449b-5p | human | nasal epithelial cells | overexpression | Marcet, et al. 2011 |
| 59 | hsa-miR-449a | human | nasal epithelial cells | overexpression | Marcet, et al. 2011 |
| 60 | hsa-miR-34c-5p | human | nasal epithelial cells | overexpression | Marcet, et al. 2011 |
| 61 | hsa-miR-34b-5p | human | nasal epithelial cells | overexpression | Marcet, et al. 2011 |
| 62 | hsa-miR-34a-5p | human | nasal epithelial cells | overexpression | Marcet, et al. 2011 |
| 63 | hsa-miR-214-3p | human | H929 | overexpression | Misiewicz-Krzeminska, et al. 2013 |
| 64 | hsa-miR-200c-3p | human | MDA-MB-231 | overexpression | Luo, et al. 2013 |
| 65 | hsa-miR-205-5p | human | MDA-MB-231 | overexpression | Luo, et al. 2013 |
| 66 | hsa-miR-375 | human | MDA-MB-231 | overexpression | Luo, et al. 2013 |
| 67 | hsa-miR-214-3p | human | U-1810 | antagomir | Salim, et al. 2013 |
| 68 | hsa-miR-103a-3p | human | H4 | overexpression | Nelson, et al. 2011 |
| 69 | hsa-miR-107 | human | H4 | overexpression | Nelson, et al. 2011 |
| 70 | hsa-miR-15b-3p | human | H4 | overexpression | Nelson, et al. 2011 |
| 71 | hsa-miR-16-5p | human | H4 | overexpression | Nelson, et al. 2011 |
| 72 | hsa-miR-195-5p | human | H4 | overexpression | Nelson, et al. 2011 |
| 73 | hsa-miR-320b | human | H4 | overexpression | Nelson, et al. 2011 |
| 74 | hsa-miR-29a-3p | human | dermal fibroblasts | inhibitor | Zhang, et al. 2013 |
| 75 | hsa-miR-224-5p | human | PC3 | overexpression | Kristensen, et al. 2014 |
| 76 | hsa-miR-452-5p | human | PC3 | overexpression | Kristensen, et al. 2014 |
| 77 | hsa-miR-224-5p | human | DU145 | overexpression | Kristensen, et al. 2014 |
| 78 | hsa-miR-452-5p | human | DU145 | overexpression | Kristensen, et al. 2014 |
| 79 | hsa-miR-34b-5p | human | hct116 | overexpression | Toyota, et al. 2008 |
| 80 | hsa-miR-34c-5p | human | hct116 | overexpression | Toyota, et al. 2008 |
| 81 | hsa-miR-100-5p | human | HUVEC | overexpression | Grundmann, et al. 2011 |
| 82 | hsa-miR-148b-3p | human | MDA-MB-231 | overexpression | Cimino, et al. 2013 |
| 83 | hsa-miR-142-3p | human | Raji | overexpression | Danger, et al. 2012 |
| 84 | hsa-miR-302a-3p | human | PC3 | overexpression | Mazda, et al. 2011 |
| 85 | hsa-miR-372-3p | human | PC3 | overexpression | Mazda, et al. 2011 |
| 86 | hsa-miR-373-5p | human | PC3 | overexpression | Mazda, et al. 2011 |
| 87 | hsa-miR-520c-3p | human | PC3 | overexpression | Mazda, et al. 2011 |
| 88 | hsa-miR-520f-3p | human | PC3 | overexpression | Mazda, et al. 2011 |
| 89 | hsa-let-7d-5p | human | fetal lung fibroblast | overexpression | Huleihel, et al. 2014 |
| 90 | hsa-miR-30b-5p | human | fetal lung fibroblast | overexpression | Huleihel, et al. 2014 |
| 91 | hsa-miR-146a-5p | human | HEK293T | overexpression | Guduric-Fuchs, et al. 2012 |
| 92 | hsa-miR-146a-5p | human | HEK293T | EVs | Guduric-Fuchs, et al. 2012 |
| 93 | hsa-miR-195-5p | human | HepG2 | overexpression | Furuta, et al. 2013 |
| 94 | hsa-miR-497-5p | human | HepG2 | overexpression | Furuta, et al. 2013 |
| 95 | hsa-miR-137 | human | U251 | overexpression | Tamim, et al. 2014 |
| 96 | hsa-miR-137 | human | U343 | overexpression | Tamim, et al. 2014 |
| 97 | hsa-miR-124-3p | human | hela | overexpression | Nam, et al. 2014 |
| 98 | hsa-miR-155-5p | human | hela | overexpression | Nam, et al. 2014 |
| 99 | hsa-miR-124-3p | human | hek293 | overexpression | Nam, et al. 2014 |
| 100 | hsa-miR-155-5p | human | hek293 | overexpression | Nam, et al. 2014 |
| 101 | hsa-miR-124-3p | human | Huh7 | overexpression | Nam, et al. 2014 |
| 102 | hsa-miR-155-5p | human | Huh7 | overexpression | Nam, et al. 2014 |
| 103 | hsa-miR-124-3p | human | imr90 | overexpression | Nam, et al. 2014 |
| 104 | hsa-miR-155-5p | human | imr90 | overexpression | Nam, et al. 2014 |
| 105 | hsa-miR-141-3p | human | SK-BR-3 | KO | Kim, et al. 2013 |
| 106 | hsa-miR-200c-3p | human | SK-BR-3 | KO | Kim, et al. 2013 |
| 107 | hsa-miR-1-3p | human | HEK293T | overexpression | Eichhorn, et al. 2014 |
| 108 | hsa-miR-1-3p | human | u2os | overexpression_cytoRNA | Eichhorn, et al. 2014 |
| 109 | hsa-miR-1-3p | human | u2os | overexpression_TotalRNA | Eichhorn, et al. 2014 |
| 110 | hsa-miR-1-3p | human | u2os | overexpression_RibozeroRNA | Eichhorn, et al. 2014 |
| 111 | hsa-miR-1-3p | human | Hela | overexpression | Eichhorn, et al. 2014 |
| 112 | hsa-miR-155-5p | human | Hela | overexpression | Eichhorn, et al. 2014 |
| 113 | hsa-let-7b-5p | human | Hela | overexpression_8h | Selbach, et al. 2008 |
| 114 | hsa-let-7b-5p | human | Hela | overexpression_32h | Selbach, et al. 2008 |
| 115 | hsa-miR-1-3p | human | Hela | overexpression_8h | Selbach, et al. 2008 |
| 116 | hsa-miR-1-3p | human | Hela | overexpression_32h | Selbach, et al. 2008 |
| 117 | hsa-miR-16-5p | human | Hela | overexpression_8h | Selbach, et al. 2008 |
| 118 | hsa-miR-16-5p | human | Hela | overexpression_32h | Selbach, et al. 2008 |
| 119 | hsa-miR-30a-5p | human | Hela | overexpression_8h | Selbach, et al. 2008 |
| 120 | hsa-miR-30a-5p | human | Hela | overexpression_32h | Selbach, et al. 2008 |
| 121 | hsa-miR-155-5p | human | Hela | overexpression_8h | Selbach, et al. 2008 |
| 122 | hsa-miR-155-5p | human | Hela | overexpression_32h | Selbach, et al. 2008 |
| 123 | hsa-miR-34a-5p | human | HCT116 | overexpression | Chang, et al. 2007 |
| 124 | hsa-miR-21-5p | human | SK-Hep1 | anti | Wagenaar, et al. 2015 |
| 125 | hsa-miR-429 | human | HEY | overexpression | Hill, et al. 2014 |
| 126 | hsa-miR-429 | human | HCCLM3 | overexpression_mimc | Li, et al. 2015 |
| 127 | hsa-miR-429 | human | HCCLM3 | anti | Li, et al. 2015 |
| 128 | hsa-miR-95-3p | human | MCF-7 | overexpression | Frankel, et al. 2014 |
| 129 | hsa-miR-147a | human | HCT116 | overexpression | Lee, et al. 2014 |
| 130 | hsa-miR-203a-3p | human | SUM159 | overexpression | Taube, et al. 2013 |
| 131 | hsa-miR-941 | human | 293T | overexpression | Hu, et al. 2002 |
| 132 | hsa-miR-10-5p | human | U87-2M1 | overexpression | Lin, et al. 2012 |
| 133 | hsa-miR-7-5p | human | A549 | overexpression | Webster, et al. 2009 |
| 134 | hsa-miR-146a-5p | human | FF3_Jurkat | overexpression | Li, et al. 2010 |
| 135 | hsa-miR-146a-5p | human | FF3_Jurkat | knockdown | Li, et al. 2010 |
| 136 | hsa-miR-155-5p | human | HEK293 | overexpression |  |
| 137 | hsa-miR-182-5p | human | liver_metastasis_sample | anti | Huynh, et al. 2011 |
| 138 | hsa-miR-99a-5p | human | miR-125b-expressing_Reh_cells | overexpression | Akbari Moqddam, et al. 2013 |
| 139 | hsa-miR-100-5p | human | miR-125b-expressing_Reh_cells | overexpression | Akbari Moqddam, et al. 2013 |
| 140 | hsa-miR-28-5p | human | P3HR1 | overexpression_12h | Schneider, et al. 2014 |
| 141 | hsa-miR-28-5p | human | P3HR1 | overexpression_24h | Schneider, et al. 2014 |
| 142 | hsa-miR-10b-5p | human | GBM4 | inhibitor | Teplyuk, et al. 2015 |
| 143 | hsa-miR-10b-5p | human | GBM6 | inhibitor | Teplyuk, et al. 2015 |
| 144 | hsa-miR-10b-5p | human | GBM8 | inhibitor | Teplyuk, et al. 2015 |
| 145 | hsa-miR-20a-5p | human | PAG | overexpression | Greenberg, et al. 2014 |
| 146 | hsa-miR-17-5p | human | PAG | overexpression | Greenberg, et al. 2014 |
| 147 | hsa-miR-671-5p | human | MCF10A | overexpression_0h | Bossel Ben-Moshe,et al. 2012 |
| 148 | hsa-miR-671-5p | human | MCF10A | overexpression_0.5h | Bossel Ben-Moshe,et al. 2012 |
| 149 | hsa-miR-671-5p | human | MCF10A | overexpression_1h | Bossel Ben-Moshe,et al. 2012 |
| 150 | hsa-miR-671-5p | human | MCF10A | overexpression_2h | Bossel Ben-Moshe,et al. 2012 |
| 151 | hsa-miR-20a-5p | human | MCF10A | overexpression_0h | Bossel Ben-Moshe,et al. 2012 |
| 152 | hsa-miR-20a-5p | human | MCF10A | overexpression_0.5h | Bossel Ben-Moshe,et al. 2012 |
| 153 | hsa-miR-20a-5p | human | MCF10A | overexpression_1h | Bossel Ben-Moshe,et al. 2012 |
| 154 | hsa-miR-20a-5p | human | MCF10A | overexpression_2h | Bossel Ben-Moshe,et al. 2012 |
| 155 | hsa-miR-191-5p | human | HepG2 | anti | Elyakim, et al. 2010. |
| 156 | hsa-miR-29a-3p | human | IMR90 | overexpression | Cushing, et al. 2011 |
| 157 | hsa-miR-27a-3p | human | RH36 | overexpression | Tombolan, et al. 2015 |
| 158 | hsa-miR-374a-5p | human | MCF | overexpression_pre | Cai, et al. 2013 |
| 159 | hsa-miR-335-5p | human | hMSC | overexpression | Tome, et al. 2011 |
| 160 | mmu-miR-15a-3p | mouse | B/CMBA.ov | overexpression | Druz, et al. 2013 |
| 161 | mmu-miR-34a-5p | mouse | C2C12 | overexpression | Marzi, et al. 2012 |
| 162 | mmu-miR-22-3p | mouse | C2C12 | overexpression | Marzi, et al. 2012 |
| 163 | mmu-miR-365-3p | mouse | C2C12 | overexpression | Marzi, et al. 2012 |
| 164 | mmu-miR-29a-3p | mouse | C2C12 | overexpression | Marzi, et al. 2012 |
| 165 | mmu-miR-145a-5p | mouse | C2C12 | overexpression | Marzi, et al. 2012 |
| 166 | mmu-miR-1a-1-5p | mouse | C2C12 | overexpression | Marzi, et al. 2012 |
| 167 | mmu-miR-378a-3p | mouse | NIH-3T3 | overexpression | Ruckerl, et al. 2012 |
| 168 | mmu-miR-378a-3p | mouse | NIH-3T3 | inhibitor | Ruckerl, et al. 2012 |
| 169 | mmu-let-7c-5p | mouse | Dgcr8 -/- ES cells | overexpression | Melton, et al. 2010 |
| 170 | mmu-miR-294-3p | mouse | Dgcr8 -/- ES cells | overexpression | Melton, et al. 2010 |
| 171 | mmu-miR-23b-3p | mouse | MLE-12 | anti | Schulz, et al. 2013 |
| 172 | mmu-miR-30d-5p | mouse | MLE-12 | anti | Schulz, et al. 2013 |
| 173 | mmu-miR-125a-5p | mouse | MLE-12 | anti | Schulz, et al. 2013 |
| 174 | mmu-miR-337-3p | mouse | MLE-12 | overexpression | Schulz, et al. 2013 |
| 175 | mmu-miR-466a-3p | mouse | MLE-12 | overexpression | Schulz, et al. 2013 |
| 176 | mmu-miR-466d-3p | mouse | MLE-12 | overexpression | Schulz, et al. 2013 |
| 177 | mmu-miR-467c-5p | mouse | MLE-12 | overexpression | Schulz, et al. 2013 |
| 178 | mmu-miR-125b-5p | mouse | 70Z/3 | overexpression | Puissegur, et al. 2012 |
| 179 | mmu-miR-125b-5p | mouse | 18-81 | overexpression | Puissegur, et al. 2012 |
| 180 | mmu-miR-182-5p | mouse | Brown adipose tissue | inhibitor | Kim, et al. 2014 |
| 181 | mmu-miR-203-3p | mouse | Brown adipose tissue | inhibitor | Kim, et al. 2014 |
| 182 | mmu-miR-708-5p | mouse | Brown adipose tissue | inhibitor | Kim, et al. 2014 |
| 183 | mmu-miR-155-5p | mouse | Dendritic cells(immature) | KO | Dueck, et al. 2014 |
| 184 | mmu-miR-155-5p | mouse | Dendritic cells(LPS) | KO | Dueck, et al. 2014 |
| 185 | mmu-miR-155-5p | mouse | Dendritic cells(oxLDL) | KO | Dueck, et al. 2014 |
| 186 | mmu-miR-155-5p | mouse | Dendritic cells(eLDL) | KO | Dueck, et al. 2014 |
| 187 | mmu-miR-155-5p | mouse | Dendritic cells(LDL) | KO | Dueck, et al. 2014 |
| 188 | mmu-miR-155-5p | mouse | Macrophages(unstimulated) | KO | Dueck, et al. 2014 |
| 189 | mmu-miR-155-5p | mouse | Macrophages(LPS) | KO | Dueck, et al. 2014 |
| 190 | mmu-miR-155-5p | mouse | Macrophages(oxLDL) | KO | Dueck, et al. 2014 |
| 191 | mmu-miR-155-5p | mouse | Macrophages(eLDL) | KO | Dueck, et al. 2014 |
| 192 | mmu-miR-155-5p | mouse | Macrophages(LDL) | KO | Dueck, et al. 2014 |
| 193 | mmu-miR-223-3p | mouse | Neutrophils | KO | Guo, et al. 2010 |
| 194 | mmu-miR-122-5p | mouse | liver | KO | Eichhorn, et al. 2014 |
| 195 | mmu-miR-124-3p | mouse | 3T3 | overexpression_inducible_contact_inhibited_12h | Eichhorn, et al. 2014 |
| 196 | mmu-miR-124-3p | mouse | 3T3 | overexpression_inducible_contact_inhibited_10h | Eichhorn, et al. 2014 |
| 197 | mmu-miR-124-3p | mouse | 3T3 | overexpression_inducible_contact_inhibited_8h | Eichhorn, et al. 2014 |
| 198 | mmu-miR-124-3p | mouse | 3T3 | overexpression_inducible_contact_inhibited_6h | Eichhorn, et al. 2014 |
| 199 | mmu-miR-124-3p | mouse | 3T3 | overexpression_inducible_contact_inhibited_4h | Eichhorn, et al. 2014 |
| 200 | mmu-miR-1a-3p | mouse | 3T3 | overexpression_inducible_contact_inhibited_48h | Eichhorn, et al. 2014 |
| 201 | mmu-miR-1a-3p | mouse | 3T3 | overexpression_inducible_contact_inhibited_12h | Eichhorn, et al. 2014 |
| 202 | mmu-miR-1a-3p | mouse | 3T3 | overexpression_inducible_contact_inhibited_8h | Eichhorn, et al. 2014 |
| 203 | mmu-miR-1a-3p | mouse | 3T3 | overexpression_inducible_contact_inhibited_4h | Eichhorn, et al. 2014 |
| 204 | mmu-miR-155-5p | mouse | 3T3 | overexpression_inducible_contact_inhibited_12h | Eichhorn, et al. 2014 |
| 205 | mmu-miR-155-5p | mouse | 3T3 | overexpression_inducible_contact_inhibited_8h | Eichhorn, et al. 2014 |
| 206 | mmu-miR-155-5p | mouse | 3T3 | overexpression_inducible_contact_inhibited_4h | Eichhorn, et al. 2014 |
| 207 | mmu-miR-1a-3p | mouse | 3T3 | overexpression_inducible_torin_treated | Eichhorn, et al. 2014 |
| 208 | mmu-miR-1a-3p | mouse | 3T3 | overexpression_inducible_actively_dividing | Eichhorn, et al. 2014 |
| 209 | mmu-miR-155-5p | mouse | 3T3 | overexpression_inducible_torin_treated | Eichhorn, et al. 2014 |
| 210 | mmu-miR-155-5p | mouse | 3T3 | overexpression_inducible_actively_dividing | Eichhorn, et al. 2014 |
| 211 | mmu-miR-155-5p | mouse | b cell | KO_inducible_contact_inhibited_48h | Eichhorn, et al. 2014 |
| 212 | mmu-miR-155-5p | mouse | b cell | KO_inducible_contact_inhibited_8h | Eichhorn, et al. 2014 |
| 213 | mmu-miR-155-5p | mouse | b cell | KO_inducible_contact_inhibited_4h | Eichhorn, et al. 2014 |
| 214 | mmu-miR-155-5p | mouse | b cell | KO_inducible_contact_inhibited_2h | Eichhorn, et al. 2014 |
| 215 | mmu-miR-196b-5p | mouse | primary_BM | transfect | Li, et al. 2012 |
| 216 | mmu-miR-196b-5p | mouse | secondary_BM | transfect | Li, et al. 2012 |
| 217 | mmu-miR-205-5p | mouse | EB | transfect | Li, et al. 2012 |
| 218 | mmu-miR-126a-3p | mouse | pDC | KO | Agudo,et al. 2014 |
| 219 | mmu-miR-188-5p | mouse | adipogenic_BMSC | KO | Li, et al. 2015 |
| 220 | mmu-miR-188-5p | mouse | osteogenic_BMSC | KO | Li, et al. 2015 |
| 221 | mmu-miR-155-5p | mouse | BMMC | KO | Biethahn, et al. 2014 |
| 222 | mmu-miR-155-5p | mouse | CD4 | knockout | Hu, et al. 2013 |

**RPF data integrated by MtiBase.**

| No. | miRNA | Species | Cell line | Treatment | Reference |
| --- | --- | --- | --- | --- | --- |
| 1 | hsa-miR-1-3p | human | hek293T | overexpression | Eichhorn, et al. 2014 |
| 2 | hsa-miR-1-3p | human | u2os | overexpression_cytoRNA | Eichhorn, et al. 2014 |
| 3 | hsa-miR-1-3p | human | u2os | overexpression_TotalRNA | Eichhorn, et al. 2014 |
| 4 | hsa-miR-1-3p | human | u2os | overexpression_RibozeroRNA | Eichhorn, et al. 2014 |
| 5 | hsa-miR-1-3p | human | hela | overexpression | Eichhorn, et al. 2014 |
| 6 | hsa-miR-155-5p | human | hela | overexpression | Eichhorn, et al. 2014 |
| 7 | mmu-miR-223-3p | mouse | Neutrophils | KO | GUO, et al. 2010 |
| 8 | mmu-miR-122-5p | mouse | liver | KO | Eichhorn, et al. 2014 |
| 9 | mmu-miR-124-3p | mouse | 3T3 | overexpression_inducible_contact_inhibited_12h | Eichhorn, et al. 2014 |
| 10 | mmu-miR-124-3p | mouse | 3T3 | overexpression_inducible_contact_inhibited_10h | Eichhorn, et al. 2014 |
| 11 | mmu-miR-124-3p | mouse | 3T3 | overexpression_inducible_contact_inhibited_8h | Eichhorn, et al. 2014 |
| 12 | mmu-miR-124-3p | mouse | 3T3 | overexpression_inducible_contact_inhibited_6h | Eichhorn, et al. 2014 |
| 13 | mmu-miR-124-3p | mouse | 3T3 | overexpression_inducible_contact_inhibited_4h | Eichhorn, et al. 2014 |
| 14 | mmu-miR-1a-3p | mouse | 3T3 | overexpression_inducible_contact_inhibited_48h | Eichhorn, et al. 2014 |
| 15 | mmu-miR-1a-3p | mouse | 3T3 | overexpression_inducible_contact_inhibited_12h | Eichhorn, et al. 2014 |
| 16 | mmu-miR-1a-3p | mouse | 3T3 | overexpression_inducible_contact_inhibited_8h | Eichhorn, et al. 2014 |
| 17 | mmu-miR-1a-3p | mouse | 3T3 | overexpression_inducible_contact_inhibited_4h | Eichhorn, et al. 2014 |
| 18 | mmu-miR-155-5p | mouse | 3T3 | overexpression_inducible_contact_inhibited_12h | Eichhorn, et al. 2014 |
| 19 | mmu-miR-155-5p | mouse | 3T3 | overexpression_inducible_contact_inhibited_8h | Eichhorn, et al. 2014 |
| 20 | mmu-miR-155-5p | mouse | 3T3 | overexpression_inducible_contact_inhibited_4h | Eichhorn, et al. 2014 |
| 21 | mmu-miR-1a-3p | mouse | 3T3 | overexpression_inducible_torin_treated | Eichhorn, et al. 2014 |
| 22 | mmu-miR-1a-3p | mouse | 3T3 | overexpression_inducible_actively_dividing | Eichhorn, et al. 2014 |
| 23 | mmu-miR-155-5p | mouse | 3T3 | overexpression_inducible_torin_treated | Eichhorn, et al. 2014 |
| 24 | mmu-miR-155-5p | mouse | 3T3 | overexpression_inducible_actively_dividing | Eichhorn, et al. 2014 |
| 25 | mmu-miR-155-5p | mouse | b cell | KO_inducible_contact_inhibited_48h | Eichhorn, et al. 2014 |
| 26 | mmu-miR-155-5p | mouse | b cell | KO_inducible_contact_inhibited_8h | Eichhorn, et al. 2014 |
| 27 | mmu-miR-155-5p | mouse | b cell | KO_inducible_contact_inhibited_4h | Eichhorn, et al. 2014 |
| 28 | mmu-miR-155-5p | mouse | b cell | KO_inducible_contact_inhibited_2h | Eichhorn, et al. 2014 |

**pSILAC data integrated by MtiBase.**

| No. | miRNA | Cell line | Treatment | Reference |
| --- | --- | --- | --- | --- |
| 1 | hsa-let-7b-5p | Hela | overexpression | Selbach, et al. 2008 |
| 2 | hsa-miR-1-3p | Hela | overexpression | Selbach, et al. 2008 |
| 3 | hsa-miR-16-5p | Hela | overexpression | Selbach, et al. 2008 |
| 4 | hsa-miR-30a-5p | Hela | overexpression | Selbach, et al. 2008 |
| 5 | hsa-miR-155-5p | Hela | overexpression | Selbach, et al. 2008 |
| 6 | hsa-let-7b-5p | Hela | knockdown | Selbach, et al. 2008 |

**Ago CLIP-Seq data integrated by Mtibase**

| GEO ID | AGO type | Species | Cell line | Method | Number | Reference |
| --- | --- | --- | --- | --- | --- | --- |
| GSE41272 | AGO | Human | huvec | HITS-CLIP | 2 | Balakrishnan, I., et al. 2013 |
| GSE41272 | AGO | Human | trbhmec | HITS-CLIP | 3 | Balakrishnan, I., et al. 2013 |
| GSE41272 | AGO | Human | hmsc | HITS-CLIP | 5 | Balakrishnan, I., et al. 2013 |
| GSE41272 | AGO | Human | hs5 | HITS-CLIP | 3 | Balakrishnan, I., et al. 2013 |
| GSE41272 | AGO | Human | hs27a | HITS-CLIP | 3 | Balakrishnan, I., et al. 2013 |
| Supplementary Data | AGO2 | Human | Jijoye | HITS-CLIP | 1 | Riley, K.J., et al. (2012) |
| GSE42701 | AGO2 | Human | Hela | HITS-CLIP | 2 | Xue, Y., et al. (2013) |
| GSE28865 | AGO2 | Human | HEK293 | HITS-CLIP | 4 | Kishore, et al. 2011 |
| GSE41357 | AGO2 | Human | BCBL-1 | HITS-CLIP | 3 | Haecker, I., et al. 2012 |
| GSE41357 | AGO2 | Human | BC-3 | HITS-CLIP | 3 | Haecker, I., et al. 2012 |
| GSE44404 | AGO2 | Human | 293S | HITS-CLIP | 6 | Karginov and Hannon. 2013 |
| GSE41285 | AGO2 | Mouse | CD4+_T_cells | HITS-CLIP | 1 | Loeb, G.B., et al. 2012 |
| GSE25310 | AGO2 | Mouse | mESCs | HITS-CLIP | 3 | Leung, A.K.L., et al. 2011 |
| Supplementary Data | AGO | Mouse | Brain | HITS-CLIP | 1 | Chi,et al. 2009 |
| GSE32109 | AGO2 | Human | BC-1 | PAR-CLIP | 1 | Gottwein, E., et al. 2011 |
| GSE32109 | AGO2 | Human | BC-3 | PAR-CLIP | 1 | Gottwein, E., et al. 2011 |
| GSE21918 | AGO1 | Human | HEK293 | PAR-CLIP | 1 | Hafner, M., et al. 2010 |
| GSE21918 | AGO2 | Human | HEK293 | PAR-CLIP | 3 | Hafner, M., et al. 2010 |
| GSE21918 | AGO3 | Human | HEK293 | PAR-CLIP | 1 | Hafner, M., et al. 2010 |
| GSE21918 | AGO4 | Human | HEK293 | PAR-CLIP | 1 | Hafner, M., et al. 2010 |
| GSE28865 | AGO2 | Human | HEK293 | PAR-CLIP | 2 | Kishore, et al 2011 |
| Supplementary Data | AGO2 | Human | hESCs | PAR-CLIP | 1 | Lipchina, I., et al. 2011 |
| GSE43573 | AGO1 | Human | HEK293 | PAR-CLIP | 3 | Memczak, S., et al. 2013 |
| GSE43573 | AGO2 | Human | HEK293 | PAR-CLIP | 1 | Memczak, S., et al. 2013 |
| GSE41437 | AGO2 | Human | EF3D | PAR-CLIP | 1 | Skalsky, R.L., et al. 2012 |
| GSE41437 | AGO2 | Human | LCL35 | PAR-CLIP | 1 | Skalsky, R.L., et al. 2012 |
| GSE41437 | AGO2 | Human | LCL-BAC | PAR-CLIP | 1 | Skalsky, R.L., et al. 2012 |
| GSE41437 | AGO2 | Human | LCL-BACD1 | PAR-CLIP | 1 | Skalsky, R.L., et al. 2012 |
| GSE41437 | AGO2 | Human | LCL-BACD3 | PAR-CLIP | 1 | Skalsky, R.L., et al. 2012 |
| Supplementary Data S1_S2_S3 | AGO1 | Human | Flp-In T-REx 293-PTH-AGO1 | CLASH | 1 | Helwak, A., et al. 2013 |

**Supplemental references**

1. Sander, S., Bullinger, L., Klapproth, K.*, et al.* (2008) MYC stimulates EZH2 expression by repression of its negative regulator miR-26a. *Blood*, **112**, 4202-4212.

2. Hausser, J., Landthaler, M., Jaskiewicz, L.*, et al.* (2009) Relative contribution of sequence and structure features to the mRNA binding of Argonaute/EIF2C-miRNA complexes and the degradation of miRNA targets. *Genome Res*, **19**, 2009-2020.

3. Hsu, P.Y., Deatherage, D.E., Rodriguez, B.A.*, et al.* (2009) Xenoestrogen-induced epigenetic repression of microRNA-9-3 in breast epithelial cells. *Cancer Res*, **69**, 5936-5945.

4. Navarro, F., Gutman, D., Meire, E.*, et al.* (2009) miR-34a contributes to megakaryocytic differentiation of K562 cells independently of p53. *Blood*, **114**, 2181-2192.

5. Fasanaro, P., Greco, S., Lorenzi, M.*, et al.* (2009) An integrated approach for experimental target identification of hypoxia-induced miR-210. *J Biol Chem*, **284**, 35134-35143.

6. Boll, K., Reiche, K., Kasack, K.*, et al.* (2013) MiR-130a, miR-203 and miR-205 jointly repress key oncogenic pathways and are downregulated in prostate carcinoma. *Oncogene*, **32**, 277-285.

7. Gregersen, L.H., Jacobsen, A.B., Frankel, L.B.*, et al.* (2010) MicroRNA-145 targets YES and STAT1 in colon cancer cells. *Plos One*, **5**, e8836.

8. Chen, A.J., Paik, J.H., Zhang, H.*, et al.* (2012) STAR RNA-binding protein Quaking suppresses cancer via stabilization of specific miRNA. *Genes Dev*, **26**, 1459-1472.

9. Rao, X., Di Leva, G., Li, M.*, et al.* (2011) MicroRNA-221/222 confers breast cancer fulvestrant resistance by regulating multiple signaling pathways. *Oncogene*, **30**, 1082-1097.

10. Shahab, S.W., Matyunina, L.V., Mezencev, R.*, et al.* (2011) Evidence for the complexity of microRNA-mediated regulation in ovarian cancer: a systems approach. *Plos One*, **6**, e22508.

11. Gaziel-Sovran, A., Segura, M.F., Di Micco, R.*, et al.* (2011) miR-30b/30d regulation of GalNAc transferases enhances invasion and immunosuppression during metastasis. *Cancer Cell*, **20**, 104-118.

12. Frankel, L.B., Wen, J.Y., Lees, M.*, et al.* (2011) microRNA-101 is a potent inhibitor of autophagy. *Embo J*, **30**, 4628-4641.

13. Hudson, R.S., Yi, M., Esposito, D.*, et al.* (2012) MicroRNA-1 is a candidate tumor suppressor and prognostic marker in human prostate cancer. *Nucleic Acids Res*, **40**, 3689-3703.

14. Setty, M., Helmy, K., Khan, A.A.*, et al.* (2012) Inferring transcriptional and microRNA-mediated regulatory programs in glioblastoma. *Mol Syst Biol*, **8**, 605.

15. Gregersen, L.H., Jacobsen, A., Frankel, L.B.*, et al.* (2012) MicroRNA-143 down-regulates Hexokinase 2 in colon cancer cells. *BMC Cancer*, **12**, 232.

16. Choudhury, Y., Tay, F.C., Lam, D.H.*, et al.* (2012) Attenuated adenosine-to-inosine editing of microRNA-376a* promotes invasiveness of glioblastoma cells. *J Clin Invest*, **122**, 4059-4076.

17. Cao, J., Shen, Y., Zhu, L.*, et al.* (2012) miR-129-3p controls cilia assembly by regulating CP110 and actin dynamics. *Nat Cell Biol*, **14**, 697-706.

18. Hudson, R.S., Yi, M., Esposito, D.*, et al.* (2013) MicroRNA-106b-25 cluster expression is associated with early disease recurrence and targets caspase-7 and focal adhesion in human prostate cancer. *Oncogene*, **32**, 4139-4147.

19. Hu, H.Y., He, L., Fominykh, K.*, et al.* (2012) Evolution of the human-specific microRNA miR-941. *Nat Commun*, **3**, 1145.

20. Shirasaki, T., Honda, M., Shimakami, T.*, et al.* (2013) MicroRNA-27a Regulates Lipid Metabolism and Inhibits Hepatitis C Virus Replication in Human Hepatoma Cells. *J Virol*, **87**, 5270-5286.

21. Hwang, W.L., Jiang, J.K., Yang, S.H.*, et al.* (2014) MicroRNA-146a directs the symmetric division of Snail-dominant colorectal cancer stem cells. *Nat Cell Biol*, **16**, 268-280.

22. Liu, M., Roth, A., Yu, M.*, et al.* (2013) The IGF2 intronic miR-483 selectively enhances transcription from IGF2 fetal promoters and enhances tumorigenesis. *Genes Dev*, **27**, 2543-2548.

23. Kiga, K., Mimuro, H., Suzuki, M.*, et al.* (2014) Epigenetic silencing of miR-210 increases the proliferation of gastric epithelium during chronic Helicobacter pylori infection. *Nat Commun*, **5**, 4497.

24. Wang, X. (2006) Systematic identification of microRNA functions by combining target prediction and expression profiling. *Nucleic Acids Res*, **34**, 1646-1652.

25. Tavazoie, S.F., Alarcon, C., Oskarsson, T.*, et al.* (2008) Endogenous human microRNAs that suppress breast cancer metastasis. *Nature*, **451**, 147-U143.

26. Ramachandran, S., Karp, P.H., Jiang, P.*, et al.* (2012) A microRNA network regulates expression and biosynthesis of wild-type and DeltaF508 mutant cystic fibrosis transmembrane conductance regulator. *Proc Natl Acad Sci U S A*, **109**, 13362-13367.

27. Fish, J.E., Santoro, M.M., Morton, S.U.*, et al.* (2008) miR-126 regulates angiogenic signaling and vascular integrity. *Dev Cell*, **15**, 272-284.

28. Cushing, L., Kuang, P.P., Qian, J.*, et al.* (2011) miR-29 Is a Major Regulator of Genes Associated with Pulmonary Fibrosis. *Am J Resp Cell Mol*, **45**, 287-294.

29. Toffanin, S., Hoshida, Y., Lachenmayer, A.*, et al.* (2011) MicroRNA-based classification of hepatocellular carcinoma and oncogenic role of miR-517a. *Gastroenterology*, **140**, 1618-1628 e1616.

30. Marcet, B., Chevalier, B., Luxardi, G.*, et al.* (2011) Control of vertebrate multiciliogenesis by miR-449 through direct repression of the Delta/Notch pathway. *Nature Cell Biology*, **13**, 693-U157.

31. Misiewicz-Krzeminska, I., Sarasquete, M.E., Quwaider, D.*, et al.* (2013) Restoration of microRNA-214 expression reduces growth of myeloma cells through positive regulation of P53 and inhibition of DNA replication. *Haematologica*, **98**, 640-648.

32. Luo, D., Wilson, J.M., Harvel, N.*, et al.* (2013) A systematic evaluation of miRNA:mRNA interactions involved in the migration and invasion of breast cancer cells. *J Transl Med*, **11**, 57.

33. Salim, H., Arvanitis, A., de Petris, L.*, et al.* (2013) miRNA-214 is related to invasiveness of human non-small cell lung cancer and directly regulates alpha protein kinase 2 expression. *Gene Chromosome Canc*, **52**, 895-911.

34. Nelson, P.T., Wang, W.X., Mao, G.*, et al.* (2011) Specific sequence determinants of miR-15/107 microRNA gene group targets. *Nucleic Acids Res*, **39**, 8163-8172.

35. Zhang, P., Huang, B., Xu, X.*, et al.* (2013) Ten-eleven translocation (Tet) and thymine DNA glycosylase (TDG), components of the demethylation pathway, are direct targets of miRNA-29a. *Biochem Biophys Res Commun*, **437**, 368-373.

36. Kristensen, H., Haldrup, C., Strand, S.*, et al.* (2014) Hypermethylation of the GABRE~miR-452~miR-224 promoter in prostate cancer predicts biochemical recurrence after radical prostatectomy. *Clin Cancer Res*, **20**, 2169-2181.

37. Toyota, M., Suzuki, H., Sasaki, Y.*, et al.* (2008) Epigenetic silencing of microRNA-34b/c and B-cell translocation gene 4 is associated with CpG island methylation in colorectal cancer. *Cancer Res*, **68**, 4123-4132.

38. Grundmann, S., Hans, F.P., Kinniry, S.*, et al.* (2011) MicroRNA-100 regulates neovascularization by suppression of mammalian target of rapamycin in endothelial and vascular smooth muscle cells. *Circulation*, **123**, 999-1009.

39. Cimino, D., De Pitta, C., Orso, F.*, et al.* (2013) miR148b is a major coordinator of breast cancer progression in a relapse-associated microRNA signature by targeting ITGA5, ROCK1, PIK3CA, NRAS, and CSF1. *Faseb J*, **27**, 1223-1235.

40. Danger, R., Pallier, A., Giral, M.*, et al.* (2012) Upregulation of miR-142-3p in peripheral blood mononuclear cells of operationally tolerant patients with a renal transplant. *J Am Soc Nephrol*, **23**, 597-606.

41. Mazda, M., Nishi, K., Naito, Y.*, et al.* (2011) E-Cadherin Is Transcriptionally Activated via Suppression of ZEB1 Transcriptional Repressor by Small RNA-Mediated Gene Silencing. *Plos One*, **6**.

42. Huleihel, L., Ben-Yehudah, A., Milosevic, J.*, et al.* (2014) Let-7d microRNA affects mesenchymal phenotypic properties of lung fibroblasts. *Am J Physiol Lung Cell Mol Physiol*, **306**, L534-542.

43. Guduric-Fuchs, J., O'Connor, A., Camp, B.*, et al.* (2012) Selective extracellular vesicle-mediated export of an overlapping set of microRNAs from multiple cell types. *BMC Genomics*, **13**, 357.

44. Furuta, M., Kozaki, K., Tanimoto, K.*, et al.* (2013) The tumor-suppressive miR-497-195 cluster targets multiple cell-cycle regulators in hepatocellular carcinoma. *Plos One*, **8**, e60155.

45. Tamim, S., Vo, D.T., Uren, P.J.*, et al.* (2014) Genomic analyses reveal broad impact of miR-137 on genes associated with malignant transformation and neuronal differentiation in glioblastoma cells. *Plos One*, **9**, e85591.

46. Nam, J.W., Rissland, O.S., Koppstein, D.*, et al.* (2014) Global analyses of the effect of different cellular contexts on microRNA targeting. *Mol Cell*, **53**, 1031-1043.

47. Kim, Y.K., Wee, G., Park, J.*, et al.* (2013) TALEN-based knockout library for human microRNAs. *Nat Struct Mol Biol*, **20**, 1458-1464.

48. Eichhorn, S.W., Guo, H.L., McGeary, S.E.*, et al.* (2014) mRNA Destabilization Is the Dominant Effect of Mammalian MicroRNAs by the Time Substantial Repression Ensues. *Mol Cell*, **56**, 104-115.

49. Selbach, M., Schwanhausser, B., Thierfelder, N.*, et al.* (2008) Widespread changes in protein synthesis induced by microRNAs. *Nature*, **455**, 58-63.

50. Chang, T.C., Wentzel, E.A., Kent, O.A.*, et al.* (2007) Transactivation of miR-34a by p53 broadly influences gene expression and promotes apoptosis. *Mol Cell*, **26**, 745-752.

51. Wagenaar, T.R., Zabludoff, S., Ahn, S.M.*, et al.* (2015) Anti-miR-21 Suppresses Hepatocellular Carcinoma Growth via Broad Transcriptional Network Deregulation. *Mol Cancer Res*, **13**, 1009-1021.

52. Hill, C.G., Jabbari, N., Matyunina, L.V.*, et al.* (2014) Functional and evolutionary significance of human microRNA seed region mutations. *Plos One*, **9**, e115241.

53. Li, L., Tang, J., Zhang, B.*, et al.* (2015) Epigenetic modification of MiR-429 promotes liver tumour-initiating cell properties by targeting Rb binding protein 4. *Gut*, **64**, 156-167.

54. Frankel, L.B., Di Malta, C., Wen, J.*, et al.* (2014) A non-conserved miRNA regulates lysosomal function and impacts on a human lysosomal storage disorder. *Nat Commun*, **5**, 5840.

55. Lee, C.G., McCarthy, S., Gruidl, M.*, et al.* (2014) MicroRNA-147 induces a mesenchymal-to-epithelial transition (MET) and reverses EGFR inhibitor resistance. *Plos One*, **9**, e84597.

56. Taube, J.H., Malouf, G.G., Lu, E.*, et al.* (2013) Epigenetic silencing of microRNA-203 is required for EMT and cancer stem cell properties. *Sci Rep*, **3**, 2687.

57. Lin, J., Teo, S., Lam, D.H.*, et al.* (2012) MicroRNA-10b pleiotropically regulates invasion, angiogenicity and apoptosis of tumor cells resembling mesenchymal subtype of glioblastoma multiforme. *Cell Death Dis*, **3**, e398.

58. Webster, R.J., Giles, K.M., Price, K.J.*, et al.* (2009) Regulation of epidermal growth factor receptor signaling in human cancer cells by microRNA-7. *J Biol Chem*, **284**, 5731-5741.

59. Li, J., Wan, Y., Guo, Q.*, et al.* (2010) Altered microRNA expression profile with miR-146a upregulation in CD4+ T cells from patients with rheumatoid arthritis. *Arthritis Res Ther*, **12**, R81.

60. Skalsky, R.L., Samols, M.A., Plaisance, K.B.*, et al.* (2007) Kaposi's sarcoma-associated herpesvirus encodes an ortholog of miR-155. *J Virol*, **81**, 12836-12845.

61. Huynh, C., Segura, M.F., Gaziel-Sovran, A.*, et al.* (2011) Efficient in vivo microRNA targeting of liver metastasis. *Oncogene*, **30**, 1481-1488.

62. Akbari Moqadam, F., Lange-Turenhout, E.A., Aries, I.M.*, et al.* (2013) MiR-125b, miR-100 and miR-99a co-regulate vincristine resistance in childhood acute lymphoblastic leukemia. *Leuk Res*, **37**, 1315-1321.

63. Schneider, C., Setty, M., Holmes, A.B.*, et al.* (2014) MicroRNA 28 controls cell proliferation and is down-regulated in B-cell lymphomas. *Proc Natl Acad Sci U S A*, **111**, 8185-8190.

64. Teplyuk, N.M., Uhlmann, E.J., Wong, A.H.*, et al.* (2015) MicroRNA-10b inhibition reduces E2F1-mediated transcription and miR-15/16 activity in glioblastoma. *Oncotarget*, **6**, 3770-3783.

65. Greenberg, E., Hajdu, S., Nemlich, Y.*, et al.* (2014) Differential regulation of aggressive features in melanoma cells by members of the miR-17-92 complex. *Open Biol*, **4**, 140030.

66. Bossel Ben-Moshe, N., Avraham, R., Kedmi, M.*, et al.* (2012) Context-specific microRNA analysis: identification of functional microRNAs and their mRNA targets. *Nucleic Acids Res*, **40**, 10614-10627.

67. Elyakim, E., Sitbon, E., Faerman, A.*, et al.* (2010) hsa-miR-191 is a candidate oncogene target for hepatocellular carcinoma therapy. *Cancer Res*, **70**, 8077-8087.

68. Cushing, L., Kuang, P.P., Qian, J.*, et al.* (2011) miR-29 is a major regulator of genes associated with pulmonary fibrosis. *Am J Respir Cell Mol Biol*, **45**, 287-294.

69. Tombolan, L., Zampini, M., Casara, S.*, et al.* (2015) MicroRNA-27a Contributes to Rhabdomyosarcoma Cell Proliferation by Suppressing RARA and RXRA. *Plos One*, **10**, e0125171.

70. Cai, J., Guan, H., Fang, L.*, et al.* (2013) MicroRNA-374a activates Wnt/beta-catenin signaling to promote breast cancer metastasis. *J Clin Invest*, **123**, 566-579.

71. Tome, M., Lopez-Romero, P., Albo, C.*, et al.* (2011) miR-335 orchestrates cell proliferation, migration and differentiation in human mesenchymal stem cells. *Cell Death Differ*, **18**, 985-995.

72. Druz, A., Chen, Y.C., Guha, R.*, et al.* (2013) Large-scale screening identifies a novel microRNA, miR-15a-3p, which induces apoptosis in human cancer cell lines. *RNA Biol*, **10**, 287-300.

73. Marzi, M.J., Puggioni, E.M., Dall'Olio, V.*, et al.* (2012) Differentiation-associated microRNAs antagonize the Rb-E2F pathway to restrict proliferation. *J Cell Biol*, **199**, 77-95.

74. Ruckerl, D., Jenkins, S.J., Laqtom, N.N.*, et al.* (2012) Induction of IL-4Ralpha-dependent microRNAs identifies PI3K/Akt signaling as essential for IL-4-driven murine macrophage proliferation in vivo. *Blood*, **120**, 2307-2316.

75. Melton, C., Judson, R.L., Blelloch, R. (2010) Opposing microRNA families regulate self-renewal in mouse embryonic stem cells. *Nature*, **463**, 621-626.

76. Schulz, M.H., Pandit, K.V., Lino Cardenas, C.L.*, et al.* (2013) Reconstructing dynamic microRNA-regulated interaction networks. *Proc Natl Acad Sci U S A*, **110**, 15686-15691.

77. Puissegur, M.P., Eichner, R., Quelen, C.*, et al.* (2012) B-cell regulator of immunoglobulin heavy-chain transcription (Bright)/ARID3a is a direct target of the oncomir microRNA-125b in progenitor B-cells. *Leukemia*, **26**, 2224-2232.

78. Kim, H.J., Cho, H., Alexander, R.*, et al.* (2014) MicroRNAs are required for the feature maintenance and differentiation of brown adipocytes. *Diabetes*, **63**, 4045-4056.

79. Dueck, A., Eichner, A., Sixt, M.*, et al.* (2014) A miR-155-dependent microRNA hierarchy in dendritic cell maturation and macrophage activation. *FEBS Lett*, **588**, 632-640.

80. Guo, H., Ingolia, N.T., Weissman, J.S.*, et al.* (2010) Mammalian microRNAs predominantly act to decrease target mRNA levels. *Nature*, **466**, 835-840.

81. Li, Z., Huang, H., Chen, P.*, et al.* (2012) miR-196b directly targets both HOXA9/MEIS1 oncogenes and FAS tumour suppressor in MLL-rearranged leukaemia. *Nat Commun*, **3**, 688.

82. Li, C., Finkelstein, D., Sherr, C.J. (2013) Arf tumor suppressor and miR-205 regulate cell adhesion and formation of extraembryonic endoderm from pluripotent stem cells. *Proc Natl Acad Sci U S A*, **110**, E1112-1121.

83. Agudo, J., Ruzo, A., Tung, N.*, et al.* (2014) The miR-126-VEGFR2 axis controls the innate response to pathogen-associated nucleic acids. *Nat Immunol*, **15**, 54-62.

84. Li, C.J., Cheng, P., Liang, M.K.*, et al.* (2015) MicroRNA-188 regulates age-related switch between osteoblast and adipocyte differentiation. *J Clin Invest*, **125**, 1509-1522.

85. Biethahn, K., Orinska, Z., Vigorito, E.*, et al.* (2014) miRNA-155 controls mast cell activation by regulating the PI3Kgamma pathway and anaphylaxis in a mouse model. *Allergy*, **69**, 752-762.

86. Hu, R., Huffaker, T.B., Kagele, D.A.*, et al.* (2013) MicroRNA-155 confers encephalogenic potential to Th17 cells by promoting effector gene expression. *J Immunol*, **190**, 5972-5980.

87. Balakrishnan, I., Yang, X., Brown, J.*, et al.* (2014) Genome-wide analysis of miRNA-mRNA interactions in marrow stromal cells. *Stem Cells*, **32**, 662-673.

88. Riley, K.J., Rabinowitz, G.S., Yario, T.A.*, et al.* (2012) EBV and human microRNAs co-target oncogenic and apoptotic viral and human genes during latency. *Embo J*, **31**, 2207-2221.

89. Xue, Y., Zhou, Y., Wu, T.*, et al.* (2009) Genome-wide analysis of PTB-RNA interactions reveals a strategy used by the general splicing repressor to modulate exon inclusion or skipping. *Mol Cell*, **36**, 996-1006.

90. Kishore, S., Jaskiewicz, L., Burger, L.*, et al.* (2011) A quantitative analysis of CLIP methods for identifying binding sites of RNA-binding proteins. *Nat Methods*, **8**, 559-564.

91. Haecker, I., Gay, L.A., Yang, Y.*, et al.* (2012) Ago HITS-CLIP expands understanding of Kaposi's sarcoma-associated herpesvirus miRNA function in primary effusion lymphomas. *PLoS Pathog*, **8**, e1002884.

92. Karginov, F.V., Hannon, G.J. (2013) Remodeling of Ago2-mRNA interactions upon cellular stress reflects miRNA complementarity and correlates with altered translation rates. *Gene Dev*, **27**, 1624-1632.

93. Loeb, G.B., Khan, A.A., Canner, D.*, et al.* (2012) Transcriptome-wide miR-155 binding map reveals widespread noncanonical microRNA targeting. *Mol Cell*, **48**, 760-770.

94. Leung, A.K., Young, A.G., Bhutkar, A.*, et al.* (2011) Genome-wide identification of Ago2 binding sites from mouse embryonic stem cells with and without mature microRNAs. *Nat Struct Mol Biol*, **18**, 237-244.

95. Chi, S.W., Zang, J.B., Mele, A.*, et al.* (2009) Argonaute HITS-CLIP decodes microRNA-mRNA interaction maps. *Nature*, **460**, 479-486.

96. Gottwein, E., Corcoran, D.L., Mukherjee, N.*, et al.* (2011) Viral microRNA targetome of KSHV-infected primary effusion lymphoma cell lines. *Cell Host Microbe*, **10**, 515-526.

97. Hafner, M., Landthaler, M., Burger, L.*, et al.* (2010) Transcriptome-wide identification of RNA-binding protein and microRNA target sites by PAR-CLIP. *Cell*, **141**, 129-141.

98. Lipchina, I., Elkabetz, Y., Hafner, M.*, et al.* (2011) Genome-wide identification of microRNA targets in human ES cells reveals a role for miR-302 in modulating BMP response. *Genes Dev*, **25**, 2173-2186.

99. Memczak, S., Jens, M., Elefsinioti, A.*, et al.* (2013) Circular RNAs are a large class of animal RNAs with regulatory potency. *Nature*, **495**, 333-338.

100. Helwak, A., Kudla, G., Dudnakova, T.*, et al.* (2013) Mapping the human miRNA interactome by CLASH reveals frequent noncanonical binding. *Cell*, **153**, 654-665.
